# Are the metabolomic responses to folivory of closely related plant species linked to macroevolutionary and plant–folivore coevolutionary processes?

**DOI:** 10.1002/ece3.2206

**Published:** 2016-06-02

**Authors:** Albert Rivas‐Ubach, José A. Hódar, Jordi Sardans, Jennifer E. Kyle, Young‐Mo Kim, Michal Oravec, Otmar Urban, Alex Guenther, Josep Peñuelas

**Affiliations:** ^1^ Environmental Molecular Sciences LaboratoryPacific Northwest National LaboratoryRichlandWashington99354USA; ^2^ CREAFCerdanyola del Vallès08913CataloniaSpain; ^3^ Grupo de Ecología TerrestreDepartamento de Biología Animal y EcologíaFacultad de CienciasUniversidad de Granada18071GranadaSpain; ^4^ CSICGlobal Ecology Unit CREAF‐CEAB‐CSIC‐UABCerdanyola del Vallès08913CataloniaSpain; ^5^ Biological Sciences DivisionPacific Northwest National LaboratoryRichlandWashington99354USA; ^6^ Global Change Research CentreAcademy of Sciences of the Czech RepublicBĕlidla 4aCZ‐603 00BrnoCzech Republic; ^7^ Department of Earth System ScienceUniversity of CaliforniaIrvineCalifornia92697USA

**Keywords:** Folivory, macroevolutionary history, *Pinus*, plant–insect coevolution, processionary moth

## Abstract

The debate whether the coevolution of plants and insects or macroevolutionary processes (phylogeny) is the main driver determining the arsenal of molecular defensive compounds of plants remains unresolved. Attacks by herbivorous insects affect not only the composition of defensive compounds in plants but also the entire metabolome. Metabolomes are the final products of genotypes and are constrained by macroevolutionary processes, so closely related species should have similar metabolomic compositions and may respond in similar ways to attacks by folivores. We analyzed the elemental compositions and metabolomes of needles from three closely related *Pinus* species with distant coevolutionary histories with the caterpillar of the processionary moth respond similarly to its attack. All pines had different metabolomes and metabolic responses to herbivorous attack. The metabolomic variation among the species and the responses to folivory reflected their macroevolutionary relationships, with *P*. *pinaster* having the most divergent metabolome. The concentrations of terpenes were in the attacked trees supporting the hypothesis that herbivores avoid plant individuals with higher concentrations. Our results suggest that macroevolutionary history plays important roles in the metabolomic responses of these pine species to folivory, but plant–insect coevolution probably constrains those responses. Combinations of different evolutionary factors and trade‐offs are likely responsible for the different responses of each species to folivory, which is not necessarily exclusively linked to plant–insect coevolution.

## Introduction

Coevolution between host plants and herbivorous insects is considered as one of the processes organizing Earth's biodiversity (Thompson and Cunningham 2002; Whitham et al. 2006). Plants have evolved several mechanisms to protect themselves from herbivorous insects, such as mechanisms involving physical, phenological, physiological, and chemical traits (Heil 2009; Campbell 2015). The chemical defenses of plants have been widely studied. They can be present before herbivorous attack and/or can be induced by folivores at the moment of attack (Mumm and Hilker 2006; Achotegui‐Castells et al. 2013). The induced response in plants includes the synthesis of chemical defensive compounds at a local level but also at a systemic level (Sticher et al. 1997; Heil and Bueno 2007; Heil 2009; Rivas‐Ubach et al. 2016). Understanding the ecology and evolution of defensive compounds induced by herbivores among different plant species remains a major challenge in plant–insect research (Poelman et al. 2008; Karban 2011; Carrillo‐Gavilán et al. 2015).

One of the main challenges is to determine whether the diversity of plant defensive compounds is predominantly driven by coevolution with herbivorous insects or by macroevolutionary processes, such as the phylogeny of plant species (Carrillo‐Gavilán et al. 2015; Endara et al. 2015). Carrillo‐Gavilán et al. 2015 analyzed the constitutive and induced defenses of several pine species and showed that the composition of the defenses was linked more to the macroevolutionary history of the plants than to the coevolution of the plant–insect interaction, suggesting that several aspects of the defensive phenotypes should converge in closely related plant species. Herbivores, however, may play a more crucial role in constraining the final expression of genotypes compared to other evolutionary factors (Kursar et al. 2009; Endara et al. 2015). The species of insects and the evolutionary histories of plant–insect interactions would be thus two crucial factors determining the chemical defensive composition of plants. The different interactions between plants and herbivores could explain why phylogenetically closely related species present divergent defensive molecular strategies to herbivores (Becerra 1997; Kursar et al. 2009).

The large battery of secondary molecular compounds that plants synthesize to confront herbivorous attack has been widely described (Herms and Mattson 1992; Kessler and Baldwin 2002). Studies of plant molecular responses induced by herbivorous attack have generally focused on particular families of metabolites or on particular molecular compounds (Sardans et al. 2011) such as terpenes (Kessler and Baldwin 2001; Mumm and Hilker 2006; Peñuelas and Staudt 2010; Heil 2014; Pierik et al. 2014) or phenolic compounds (Bennett and Wallsgrove 1994; Carrillo‐Gavilán et al. 2015). Metabolomic studies, however, have shown that folivory not only produces shifts in the arsenal of defensive compounds of plants but destabilizes the internal homeostasis, shifting the entire metabolome of the host plants, including both primary and secondary metabolisms (Jansen et al. 2008; Rivas‐Ubach et al. 2014, 2016).

The metabolome of an organism is the final expression of the genotype and provides information for a variety of complex physiological processes occurring in an organism at a particular moment (Fiehn 2002). The metabolome of an organism is the first to respond to environmental changes and is considered as the chemical phenotype of the organism (Peñuelas and Sardans 2009). Ecometabolomics, the use of metabolomic techniques in ecological studies (Peñuelas and Sardans 2009; Rivas‐Ubach et al. 2013), is a suitable and powerful tool for studying the metabolic responses of wild organisms under biotic or abiotic stressors and under changes of natural environmental conditions (Shulaev et al. 2008; Sardans et al. 2011; Rivas‐Ubach et al. 2012, 2016). Metabolomic techniques are sufficiently sensitive to distinguish between significant shifts of metabolomes in different varieties of the same plant species produced by attacks by the same species of insect (Mirnezhad et al. 2010). Evolutionary theories, however, propose that phylogenetically closely related species should have similar phenotypic responses to the same stressors (Blomberg et al. 2003; Carrillo‐Gavilán et al. 2015) although the phenotype of a species can strongly be modified by epigenetic modifications (Rapp and Wendel 2005) and even the microbiome (Peñuelas and Terradas 2014). By this premise, the chemical phenotypes should be more similar between closely related species than between distant related species. Whether wild populations of closely related plants having different coevolutionary histories with herbivores have similar or divergent metabolomic responses to attacks by the same folivore thus remains unclear. This information would provide important clues to determine whether metabolomic structure, including defensive compounds and other metabolites associated with folivory, is mainly due to macroevolutionary (phylogenetic) or plant–insect coevolutionary processes.

The caterpillar of the pine processionary moth (PPM), *Thaumetopoea pityocampa* (Denis & Schiffermüller), distributed throughout the Mediterranean Basin (Kerdelhué et al. 2009), is a good candidate for studying the plant–folivore relationship, because all larval stages develop on the same tree and the trees suffer continuous folivory for months from the same herbivore (Battisti et al. 2006, 2015; Jactel et al. 2015). The PPM is an oligophagous species that feeds almost exclusively on needles of *Pinus* species, although with different preferences among species (Avtzis 1986; Battisti 1988; Hódar et al. 2003; Jactel et al. 2015). PPM larvae develop during winter, so their distribution is mainly limited by winter temperatures (Huchon and Démolin 1971; Hoch et al. 2009). The expected increase in temperatures in the near future (IPCC, 2013) would provide the climatic conditions favoring the expansion of the PPM's range, thus constituting a serious problem for the conservation of several pine populations (Battisti et al. 2015), especially relictual populations (Blanca et al. 1998; Hódar et al. 2003). Several studies have reported that the PPM has recently become able to reach pine species naturally distributed at higher latitudes and altitudes due the global increase in temperatures (Benigni and Battisti 1999; Hódar et al. 2003; Battisti et al. 2005, 2006; Netherer and Schopf 2010) and are now the main folivorous pests of pine woodlands in the area covering most pine species (Battisti et al. 2015; Jactel et al. 2015).

**Figure 1:**
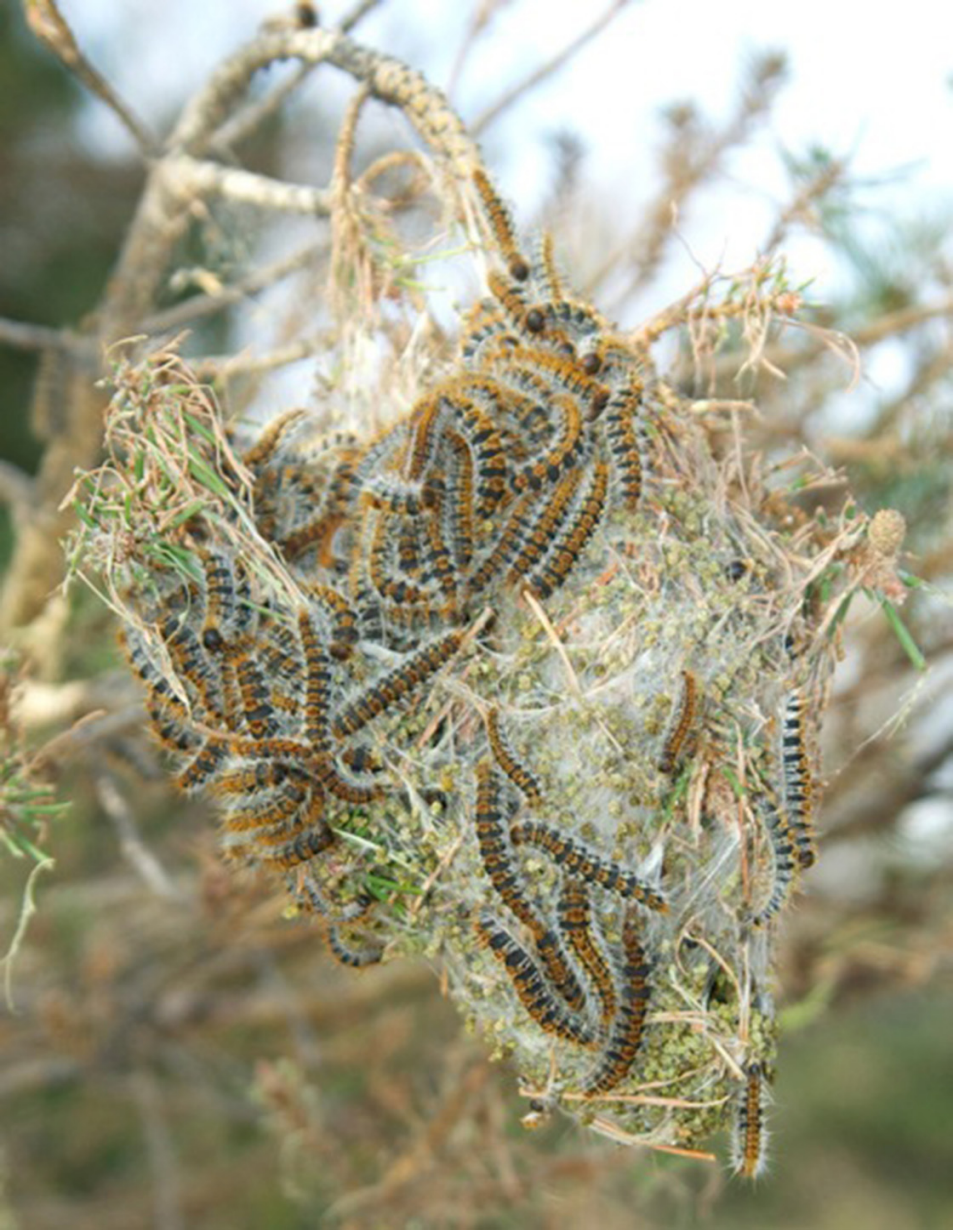
Fifth stage of caterpillars of the pine processionary moth (PPM) feeding on *P. sylvestris* needles in Lanjarón (southeast Spain) in winter 2011. The study was performed in summer so to study the responses of pines later on after the winter attack by PPM.

We used metabolomic techniques to determine how the metabolic responses of three populations of closely related pine species living in the same mountain cope with attacks by the same species of herbivorous insect, the PPM. The Quaternary history of the PPM together with the singular topography of the Iberian Peninsula has created a scenario of different plant–insect interactive situations, in which the different pine species had distinct, intense coevolutionary histories with the defoliator (Rousselet et al. 2010). In summer, we sampled needles from nonattacked trees and needles from attacked trees flushed after a folivory attack during the previous winter (Fig. 1) (NATs and ATs, respectively) of *Pinus pinaster*,* P*. *nigra,* and *P*. *sylvestris* (hereafter *pinaster*,* nigra* and *sylvestris*, respectively) and analyzed the elemental compositions and metabolomes of the needles. This study advances our understanding of the responses of plants to folivory by contrasting entire metabolomic structures, including several metabolites associated with folivory, of three closely related pine species of the Mediterranean Basin.

## Material and Methods

### Study site

Samples were collected in July 2011 (summer) at three sites in the Loma de Lanjarón in Sierra Nevada Natural and National Park (Granada SE Spain) (36°56′N, 3°28′W). Populations of *P. pinaster* were at the lowest site (Cortijo Quemado, 1350 m a.s.l.), *P*. *nigra* populations were at the mid‐altitudinal site (Cruce de Tello, 1700 m a.s.l.), and *P. sylvestris* populations were at the highest site (Peña Caballera, 2050 m a.s.l.). The climate in the park is Mediterranean with dry, hot summers typically leading to severe summer droughts and cold winters. Winter snow usually persists from November to March above 2000 m a.s.l. The mean annual precipitation is 470 ± 50 mm (±SE, 1988–2008; data are from a meteorological station at 1450 m a.s.l.), and the mean annual temperature is 12.3 ± 0.4°C at 1650 m a.s.l. (1994–2008; State Meteorological Agency, Ministry of the Environment). Along the elevational gradient, temperature decreases by approximately 0.5°C, and rainfall increases by 27 mm for every 100‐m increase in elevation. A more detailed description of the climate and soil in the area has been provided by Cuadros and Francia (1999). Each pine species thus grows under its preferred environmental conditions of temperature and humidity which allows a better understanding of the metabolomic responses of pines to the attack of the PPM in natural conditions.

### Experimental design and needle sampling

Ten trees each of *pinaster*,* nigra,* and *nevadensis* without signs of PPM folivory (NATs) were randomly sampled at each site as study subjects. NAT trees presented at least three cohorts of needles (including the new ones flushed in spring 2011) indicating that those individuals had been at least two consecutive winters without defoliation by PPM. Ten other trees of each species and similar in height and age with evident signs of defoliation by the PPM the previous winter (ATs) were also sampled. AT pines presented exclusively the most recent cohort of needles, that is, the ones flushed in spring 2011, indicating thus that those trees suffered a total defoliation during the previous winter (Fig. 1). The total number of individuals in this study was thus 60. Both the lack of previous defoliation and the recent damage of the ATs are easily recognizable by visually examining the different cohorts of needles (see Rivas‐Ubach et al. 2016 for more details of experimental design and sampling). The three sites had vigorous and healthy trees mostly from the natural regeneration of trees 10–15 years old and 2–3 m in height. Current‐year needles from several twigs were collected from each tree, placed in labeled paper envelopes, and stored quickly in liquid nitrogen. PPMs defoliate trees during winter and the collected needles were flushed in April–June, so all sampled needles, even those from the ATs, showed no sign of folivory. The analyzed metabolomes thus represented the systemic responses of trees to PPM attack. Sampling different species at different altitudes should not affect the consistency of our findings, because the different altitudes represent the most favorable climatic conditions for each of the species. Each species is adapted to the specific environmental conditions at each altitude, with their corresponding trade‐offs constrained by evolutionary processes, so we are thus comparing the natural metabolomes of three closely related populations of pines. The intensity of defoliation could vary significantly along the altitudinal gradient since it mainly depends on the climatic conditions on winter (Hódar et al. 2003, 2012;  Hódar and Zamora 2004) and the amount of predators and parasitoids that typically decrease with altitude (Hódar 2015). Our sampling, however, was based on nondefoliated (NATs) vs. defoliated pines (ATs) and ignores thus any possible effect of folivory intensity on pine metabolomes.

### Foliar processing for elemental and metabolomic analyses

The needles preserved in liquid nitrogen were lyophilized and stored in plastic containers at −20°C. Each sample was ground with a ball mill at 1600 rpm for 12 min (Mikro‐Dismembrator‐U; B. Braun Biotech International, Melsungen, Germany) to a fine homogeneous powder and stored at −80°C until the extraction of the metabolites for analysis by liquid chromatography–mass spectrometry (LC‐MS).

### Elemental analysis

For C and N determination, 1.4 mg of sample powder was analyzed with a CHNS‐O Elemental Analyser (EuroVector, Milan, Italy). P and K were extracted by acid digestion in a microwave reaction system under high temperature and pressure (Sardans et al. 2010). Briefly, 250 mg of sample powder was placed in a Teflon tube with 5 mL of nitric acid and 2 mL of H_2_O_2_. A MARSXpress microwave reaction system was used for the digestions (CEM, Mattheus, NC). The digested material was transferred to 50‐mL flasks and resuspended in Milli‐Q water to a final volume of 50 mL. P and K concentrations were determined by optic emission spectrometry with inductively coupled plasma (Perkin‐Elmer Corporation, Norwalk, CA) as described in detail in the supporting information in Rivas‐Ubach et al. (2016).

### Extraction of metabolites for LC‐MS analysis

Extracts of polar and semi‐polar metabolites were prepared as outlined by t'Kind et al. (2008), with minor modifications. Briefly, two sets of 2‐mL microcentrifuge tubes were labeled: set A for the metabolite extractions and set B for the extracts from set A. One hundred milligrams of pine powder from each sample was transferred to a 2‐mL tube of set A, and 1 mL of extractant (80:20 MeOH:H_2_O) was added. All tubes were vortexed for 15 min, sonicated for 5 min at 24°C, and then centrifuged at 23,000 × *g* for 5 min. After centrifugation, 0.6 mL of the supernatant from each tube of set A was transferred to the corresponding 2‐mL tube of set B. Each sample was then re‐extracted using the same procedure. The two extracts of each sample were combined and filtered through 0.22‐*μ*m syringe microfilters and transferred to a labeled set of high‐performance liquid chromatography (HPLC) vials. Extracts were stored at −80°C until the LC‐MS analysis.

### HPLC‐MS analysis

High‐performance liquid chromatography was performed with a reversed‐phase C18 Hypersil gold column (150 × 2.1 mm, 3‐*μ*m particle size; Thermo Scientific, Waltham, MA) and a Dionex Ultimate 3000 HPLC system (Thermo Fisher Scientific/Dionex RSLC, Dionex, Waltham, MA). The chromatograph was operated at a constant temperature of 30°C and a flow rate of 0.3 mL min^−1^. Five microliters of each sample was injected. Mobile phases consisted of water (0.1% acetic acid) (A) and acetonitrile (B). Both mobile phases A and B were previously degassed for 10 min in an ultrasonic bath. The elution gradient started with 90% A (10% B) and was maintained for 5 min, and the elution then changed linearly to 10% A (90% A) over the next 15 min. The elution gradient was then returned linearly to the initial conditions (90% A and 10% B) over the next 5 min. The chromatographic column was washed and stabilized for 5 min before the next sample was injected.

The HPLC was coupled to an LTQ Orbitrap XL high‐resolution mass spectrometer (Thermo Fisher Scientific, Waltham, MA) equipped with a HESI II (heated electrospray ionization) source for mass spectrometric analyses. All samples were injected twice, once with the HESI operating in positive ionization mode (+H) and once in negative ionization mode (−H). The mass spectrometer was operated in Fourier transform mass spectrometry full‐scan mode with high mass resolution (60,000) at a mass range of *m*/*z* 50–1000. A caffeine standard was injected every 10 samples to monitor the resolution and sensitivity of the spectrometer. The resolution was further monitored with lock masses (phthalates). Blank samples were also analyzed during the sequence. For more information for the Orbitrap parameters, see the supporting information by Rivas‐Ubach et al. (2016).

### Processing of LC‐MS chromatograms

The raw data files from the spectrometer were processed using MZmine 2.12 (Pluskal et al. 2010). The chromatograms of both the positive and negative modes were always treated separately. All chromatograms were baseline corrected, and ion chromatogram lists were generated. These ion chromatograms were subsequently deconvoluted, aligned, and autoassigned (See Table S1 for parameter details). The metabolites in each generated data set (+H and −H) were assigned by total exact mass, exact mass of fragments, and retention time (RT) from the measurements of standards in the LC‐MS Orbitrap system (Table S2). Even though the metabolite assignation was putative, the high resolution and RTs significantly decreased the number of false positives (Sumner et al. 2007). The numerical data sets were then exported as CSV files and filtered. The numerical values of the variables of the data sets correspond to the absolute peak areas of the chromatograms detected by the spectrometer. The area is directly proportional to the concentration of the variable and is suitable for comparative analyses in metabolomic studies (Lee and Fiehn 2013; Mari et al. 2013; Nuringtyas et al. 2014; Rivas‐Ubach et al. 2014). We thus use the term *concentration* when referring to differences between the metabolites, although it does not represent a true concentration as the weight of a molecular compound per weight of sample. Some ions with identical masses may finally present slightly different RTs during chromatogram generation and deconvolution, so all the indentified variables by our library that showed such characteristics variables were summed to produce only one variable per metabolite. Some groups of sugars with identical molecular masses co‐eluted at the same RT with the chromatographic method used, so we could not differentiate them at MS^1^ (first tandem in mass spectrometry). Different sugars were thus grouped by mass RT (hexoses: glucose, fructose, mannose, and galactose; pentoses: arabinose, ribose, and xylose; disaccharides: sucrose and maltose; group 1 sugars (S1): deoxyglucose, deoxygalactose, and D‐fucose; group 2 sugars (S2): xylitol and arabitol). Outliers for specific variables were removed from the data set and treated as missing data. Outliers were defined as measurements threefold higher than the third quartile or threefold lower than the first quartile of each cellular factor (each species × folivory level (FL: N‐ATs and ATs) combination). None of the assigned variables contained outliers for any of the pine individuals. Variables with fewer than six individuals in all cellular factors (species × FL) were removed from the data set.

### Statistical analyses

The study data set consisted of two categorical variables (species, with three levels, *pinaster*,* nigra,* and *sylvestris*; and FL, with two levels, NATs and ATs) and 8861 continuous variables, nine of which corresponded to elemental and stoichiometric variables (C, N, P, and K concentrations and C:N, N:P, C:P, N:K, and K:P ratios) and 8852 of which were metabolomic variables, including 49 identified by our library of plant metabolites.

To test for differences between species and FL in the overall elemental stoichiometry and metabolome, the complete data set (8861 variables) of the *pinaster*,* nigra,* and *sylvestris* pines was subjected to a permutational manova (PERMANOVA) using the *Bray–Curtis* distance. A PERMANOVA was also performed for each of the species separately to test for differences between FLs. The number of permutations was set at 10,000 for all PERMANOVAs.

The stoichiometric and metabolomic data for the three species were also subjected to principal component analysis (PCA) to determine the natural variability among the species and FLs. The score coordinates of the variables of the PCAs were subjected to one‐way ANOVAs to identify statistical differences between the species and the two FLs (See Supporting Information Rivas‐Ubach et al. 2013). Partial least square discriminant analyses (PLS‐DAs) were additionally performed for each of the species separately with FL as the discriminant factor using the complete data set (8861 variables) to determine which of the known variables most separated the FLs for each species.

Metabolomic distances (Euclidian distances) were calculated among N‐ATs of the species by using two different data inputs: (1) the N‐AT case coordinates of the first 10 PCs of the case plot of the PCA performed with the complete data set, including NATs and ATs; the weight of each of the PCs (explained variability) was considered for the calculation and (2) the complete data set, including all stoichiometric and metabolomic variables (8861 variables) of the NATs alone. These N‐AT distances were then used to plot a cluster dendrograms. All metabolomic distances for each pair of species were subsequently submitted to one‐way ANOVAs to detect differences between species.

An additional PCA was performed for all variables (8861 variables) but using the residual values of ATs relative to N‐ATs to explore the metabolomic variation of the response to folivory among the species. For the calculation of the residual value of ATs relative to N‐ATs, the mean N‐AT value for each species was calculated for each of the variables of the data set. The mean value of each variable for each species was then subtracted from the value for each AT individual of the corresponding species as detailed by the following formula:

$${\left[\text{Residual}\right]}_{{\mathrm{Ats}-\mathrm{NATs}}_{ijz}}={\left[\mathrm{AT}-\mathrm{Variable}\right]}_{ijz}-\left[\frac{\sum {\left[\mathrm{NAT}-\mathrm{Variable}\right]}_{imz}}{n_{{\mathrm{NAT}}_{iz}}}\right]$$

where ${\left[\mathrm{AT}-\mathrm{Variable}\right]}_{ijz}$ is the concentration value for the variable *i* of the attacked individual *j* of species *z;* $\sum {\left[\mathrm{NAT}-\mathrm{Variable}\right]}_{ijz}$ is the sum of all the concentration values for the variable *i* for the nonattacked individual *m* of species *z*, and $n_{{\mathrm{NAT}}_{iz}}$ is the number of individuals of the nonattacked trees for the variable *i* and species *z*.

The Euclidian distances of the residuals of ATs relative to NATs were also calculated between species, and a dendrogram was plotted.

One‐way ANOVAs were also performed for each stoichiometric and metabolite variable to identify any statistical differences between FLs for each species (ANOVAs of elemental, stoichiometric, and assigned metabolites are shown in Table S3). We first assessed the normality and homogeneity of the variances by Shapiro–Wilk and Levene's tests, respectively. All identified metabolites met the assumptions of the *F* statistic, but 124 unassigned variables of the 8861 variables did not and were thus removed from the data set. Benjamini–Hochberg corrections of the *P* values from the one‐way ANOVAs were subsequently performed for the total set of variables that met the assumptions (8737 variables).

The PERMANOVAs, PCAs, one‐way ANOVAs, Shapiro–Wilk tests, and Levene's tests were performed with R (R Core Team 2013). The Shapiro–Wilk tests, one‐way ANOVAs, Euclidian distances, and cluster dendrograms used the functions *shapiro.test, aov*,* dist,* and *hclust*, respectively, in the “stats” package (R Core Team 2013). Cluster dendrograms were plotted using the complete method. Levene's tests were performed with the *leveneTest* function of the “car” package (Fox and Weisberg 2011). The PERMANOVAs were conducted with the *adonis* function of the “vegan” package (Oksanen et al. 2013). The PCAs and PLS‐DAs were performed using the *pca* and *pls.da* functions, respectively, of the *mixOmics* package (Le Cao et al. 2015). Data were scaled for the PCAs and PLS‐DAs through the corresponding function.

## Results

The PERMANOVA including both species and FL factors identified significant differences in the elemental concentrations and their ratios and in the metabolomes of the needles of the three species (pseudo‐*F *=* *42.3; *P *<* *0.0001), identified marginally significant differences between the FLs (pseudo‐*F *=* *1.9; *P *=* *0.096) and identified significant differences in the species × FL interaction (pseudo‐*F *=* *2.7; *P *<* *0.05) (Table 1).

**Table 1:** Full PERMANOVA model including all elemental, stoichiometric, and metabolomic variables (8861 continuous variables) and the two categorical variables, species and folivory level, and their interaction

	Degrees of freedom	Sums of squares	Mean squares	Pseudo‐*F*	*P*
Species	2	3.001	1.501	42.33	0.0001
Folivory level (FL)	1	0.068	0.068	1.906	0.0990
Species × FL	2	0.194	0.097	2.742	0.0130
Residuals	54	1.915	0.035	0.370	
Total	59	5.178	1		

The PERMANOVA for each species indicated significant levels of folivory in *pinaster* (pseudo‐*F *=* *2.18; *P *<* *0.01) and *sylvestris* (pseudo‐*F *=* *4.31; *P *<* *0.001) but not *nigra* (pseudo‐*F *=* *1.45; *P *=* *0.157) (Table 2).

**Table 2:** PERMANOVAs for each species including all elemental, stoichiometric, and metabolomic variables (8861 continuous variables), with folivory level as the categorical variable

	Degrees of freedom	Sums of squares	Mean squares	Pseudo‐*F*	*P*
*Pinus pinaster*					
Folivory level	1	0.066	0.066	2.18	0.00790
Residuals	18	0.541	0.030	0.89	
Total	19	0.607	1		
*Pinus nigra*					
Folivory level	1	0.072	0.072	1.45	0.15760
Residuals	18	0.890	0.049	0.93	
Total	19	0.961	1		
*Pinus sylvestris*					
Folivory level	1	0.118	0.118	4.31	0.00001
Residuals	18	0.490	0.027	0.81	
Total	19	0.608	1		

**Figure 2:**
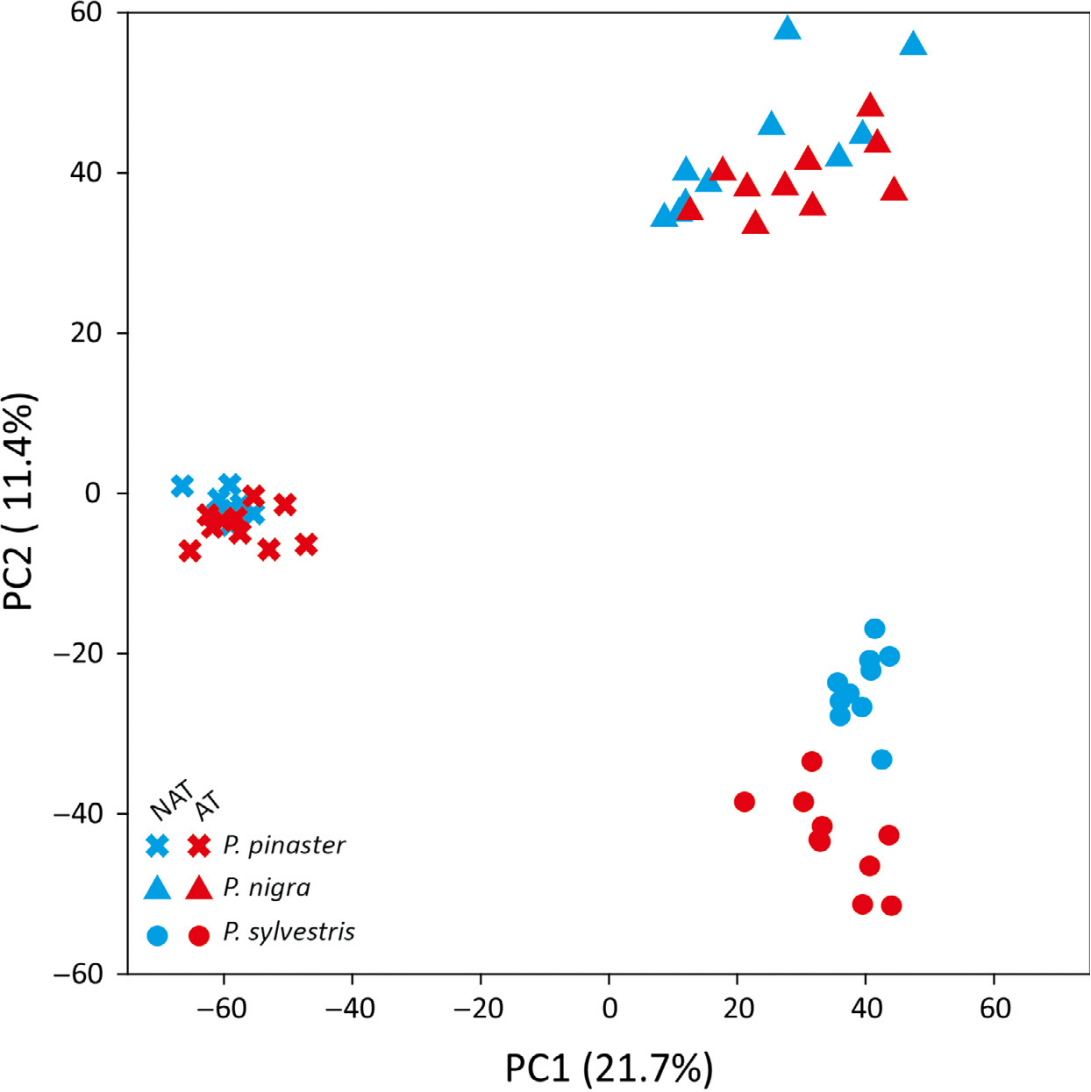
Principal component 1 (PC1) versus PC2 of the case plot of the principal component analysis (PCA) of the metabolomic and elemental composition variables for *P. pinaster*,* P. nigra,* and *P. sylvestris* species. Folivory level (FL) is represented by different colors: blue for not attacked trees (N‐ATs) and red for attacked trees (ATs). Different pine species are represented by different shape: crosses for *P. pinaster*, triangles for *P. nigra,* and circles for *P. sylvestris*.

The first 10 PCs of the PCA with all species explained 55.2% of the total stoichiometric and metabolomic variance among species (PC1 = 21.7%, PC2 = 11.4%, PC3 = 5.4%, PC4 = 3.4%, PC5 = 3.1%, PC6 = 2.4%, PC7 = 2.1%, PC8 = 2.1%, PC9 = 1.8%, PC10 = 1.78%). One‐way ANOVAs on PC1 and PC2 case coordinated showed clearly clustering of the three species (*F *=* *793 and *P *<* *0.0001 for PC1, *F *=* *499 and *P *<* *0.0001 for PC2) (Fig. 2). PC1 versus PC2 of the case plot of PCA identified *pinaster* as the most divergent pine of the three species. One‐way ANOVAs of the case coordinates for each species also identified significant differences between FLs in *pinaster* and *sylvestris* along PC2 (*F *=* *8.09 and *P *<* *0.05, *F *=* *64.8 and *P *<* *0.0001, respectively). PC1 marginally separated the FLs for *sylvestris* (*F *=* *3.23; *P *=* *0.089). The FLs did not differ significantly for *nigra* in either the PC1 or PC2 axis (*P *>* *0.05).

One‐way ANOVAs between the metabolomic distances of the N‐ATs calculated with the first 10 PCs of the PCA identified significant differences between all distance comparisons (*F *=* *1314 and *P *<* *0.00001 for *pinaster–nigra* vs *sylvestris–nigra* distances, *F *=* *1775 and *P *<* *0.00001 for *pinaster–sylvestris* vs *nigra–sylvestris* distances and *F *=* *41.0 and *P *<* *0.00001 for *pinaster–nigra* vs *pinaster–sylvestris* distances; Table 3). In contrast, metabolomic distances of the NATs calculated with the complete data set (8861 variables) identified significant differences between *pinaster–nigra* versus *sylvestris–nigra* distances (*F *=* *43.0 and *P *<* *0.00001) and between *pinaster–sylvestris* versus nigra–*sylvestris* distances (*F *=* *43.8 and *P *<* *0.00001). The *pinaster–nigra* vs *pinaster–sylvestris* distances, however, did not differ significantly when using the complete variable data set (*F *=* *0.013 and *P *=* *0.91) (Table 3).

**Table 3:** Metabolomic distances (Euclidian distances) between pairs of pine species calculated with (1) the case coordinates of the first 10 PCs of the PCA (Fig. 2) and (2) the entire metabolome. One‐way ANOVAs were applied to each combination of distances between species. The *F* and *P* values are shown

Stoichiometric and metabolomic distances between pine species	Euclidian distance of the entire metabolome		Euclidian distance of the coordinates of the first 10 PCs of the PCA	
	Mean	SE	Mean	SE
*pinaster–nigra*	4.44 × 10^9^	1.98 × 10^8^	19.22	0.207
*pinaster–sylvestris*	4.41 × 10^9^	1.89 × 10^8^	21.16	0.219
*nigra–sylvestris*	2.83 × 10^9^	1.67 × 10^8^	9.6	0.165

Comparison distances (one‐way ANOVA)	*F*	*P*	*F*	*P*
*pinaster–nigra* versus *nigra–sylvestris*	43.0	<0.00001	1314	<0.00001
*pinaster–sylvestris* versus *nigra–sylvestris*	43.8	<0.00001	1775	<0.00001
*pinaster–nigra* versus *pinaster–sylvestris*	0.013	0.910	41.0	<0.00001

**Figure 3:**
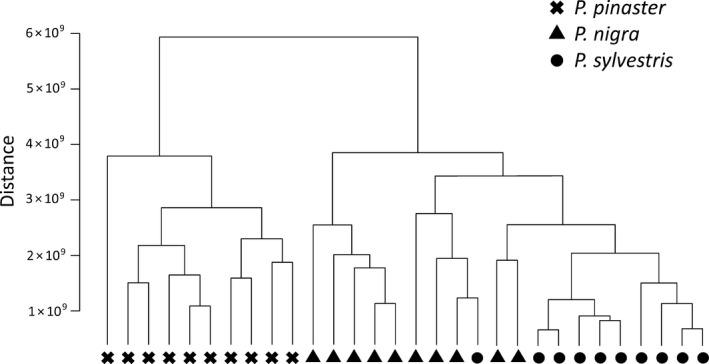
Dendrogram representing the metabolomic distances (based on Euclidian distances) between the nonattacked trees of *Pinus pinaster*,* P. nigra,* and *P. sylvestris*.

The cluster dendrogram plotted by Euclidian distances using the complete data set of N‐ATs also identified *pinaster* as the most distant pine among the three species (Fig. 3). The dendrogram identified smaller distances between *nigra* and *sylvestris*, but the two species clustered well, and *nigra* was closer than *sylvestris* to *pinaster*. Only one case of *sylvestris* was plotted within the *nigra* cluster, but the dendrogram generally identified *sylvestris* as the pine with the lowest metabolomic variability among its individuals.

**Figure 4:**
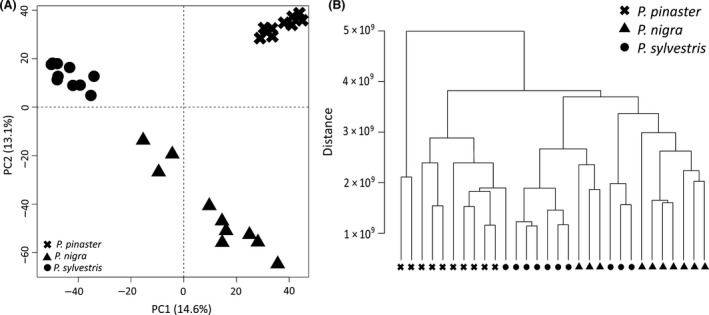
PC1 versus PC2 of the case plot of the PCA using the residuals of the ATs relative to NATs (A). Dendrogram representing the metabolomic distances (based on Euclidian distances) between the residuals of the ATs relative to NATs for *Pinus pinaster*,* P. nigra,* and *P. sylvestris* (B).

Principal component analysis performed with the residuals of ATs relative to NATs showed clear separation among *pinaster*,* nigra,* and *sylvestris* (Fig. 4A). PC1 and PC2 explained 14.6 and 13.1% of the total variance, respectively. The cluster dendrogram for the residuals of ATs relative to NATs again indicated that the metabolomic responses to folivory of *pinaster* were the most divergent than those of *nigra* and *sylvestris*, which were much more similar (Fig. 4B).

**Figure 5:**
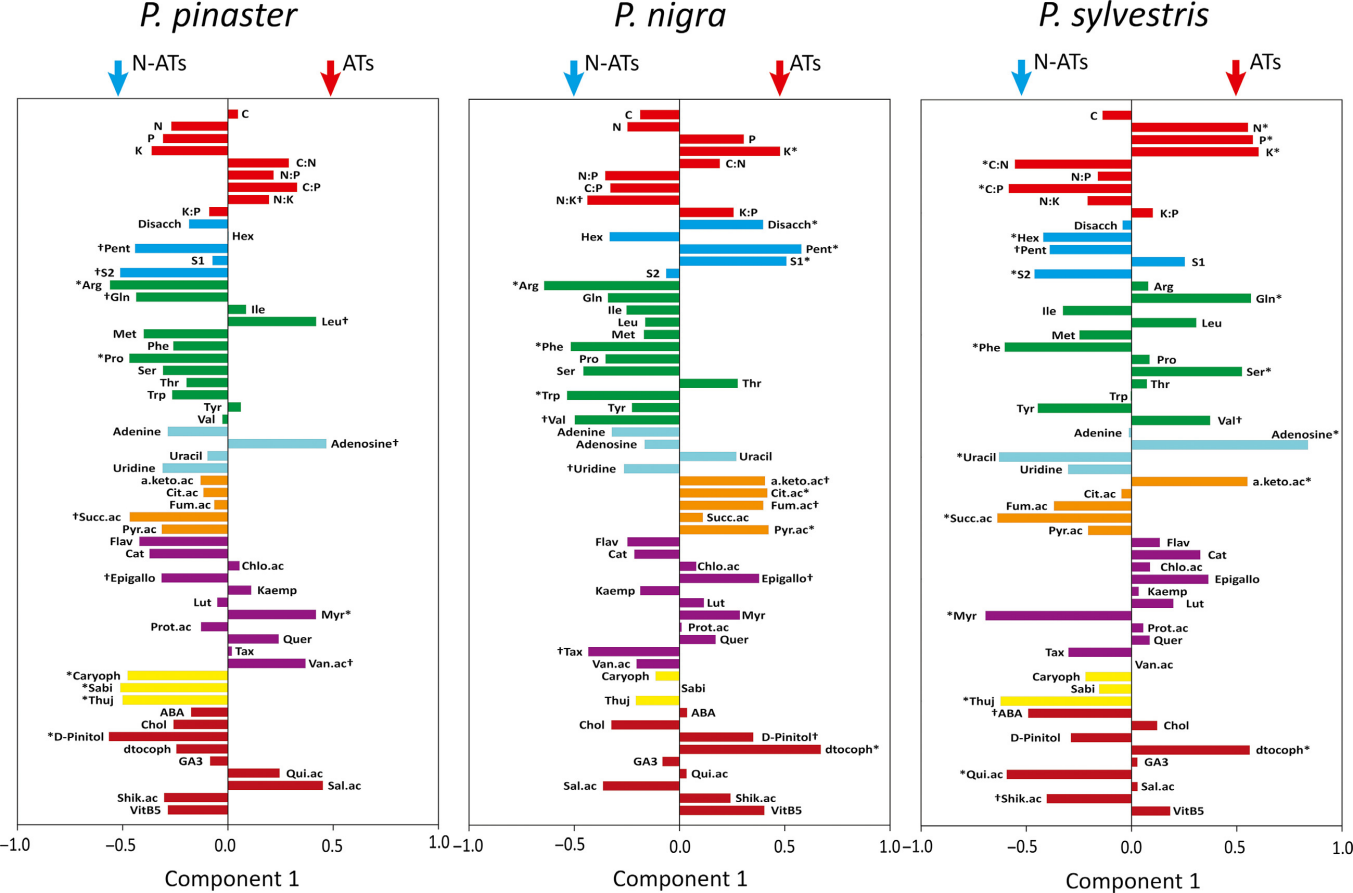
Component 1 of the plot of the partial least squares discriminant analysis (PLS‐DA) of the metabolomic and stoichiometric variables for *Pinus pinaster*,* P. nigra,* and *P. sylvestris*, with folivory level (FL) as the discriminant factor. Colored arrows above the variable plot of each species represent the mean of the distribution of the FLs of the case plot. Unassigned metabolites are not represented in the graph. Elemental and stoichiometric variables are shown in red. Metabolomic functional groups are represented by different colors: blue, sugars; green, amino acids; cyan, nucleotides; orange, organic acids associated with the Krebs cycle; violet, phenolics; yellow, terpenes; dark red, other metabolites. Most metabolites are identified by abbreviations: disaccharides (Disacch): hexoses (Hex) and pentoses (Pent); group 1 sugars: deoxyglucose, deoxygalactose, and D‐fucose (S1); group 2 sugars: xylitol and arabitol (S2); arginine (Arg); glutamine (Gln); isoleucine (Ile); leucine (Leu); methionine (Met); phenylalanine (Phe); proline (Pro); serine (Ser); threonine (Thr); tryptophan (Trp); tyrosine (Tyr); valine (Val); *α*‐ketoglutaric acid (a‐keto.ac); citric acid (Cit.ac); fumaric acid (Fum.ac); succinic acid (Succ.ac); pyruvic acid (Pyr.ac); trimethoxyflavone (Flav); catechin (Catech); chlorogenic acid (Chlo.ac); epigallocatechin (Epigalloc); kaempferol (Kaemp); luteolin (Lut); myricetin (Myr); protocatechuic acid (Prot.ac); quercetin (Quer); taxifolin (Tax); vanillic acid (Van.ac); caryophyllene (Caryoph); sabinene (Sabi); thujone (Thuj); abscisic acid (ABA); choline (Chol); *δ*‐tocopherol (dtocoph); pantothenic acid (Vit B5); quinic acid (Quin.ac); shikimic acid (Shik.ac); gibberellic acid 3 (GA3); and salicylic acid (Sal.ac). Variables with asterisks or crosses differed significantly (*P *<* *0.05) or marginally significantly (*P *<* *0.1) between FLs in one‐way ANOVAs (Table S3).

The PLS‐DA for each species with FL as the discriminant factor indicated that the pines generally responded differently to PPM attacks for the identified variables (Fig. 5, Table S3). Of the total number of variables that met the assumptions for a one‐way ANOVA (8737 variables), 1863 (21.3%), 1126 (12.9%), and 2396 (27.4%) of the variables differed significantly (*P *<* *0.05) in the one‐way ANOVAs between the FLs for *pinaster*,* nigra,* and *sylvestris*, respectively (Table 4). Benjamini–Hochberg correction of the *P* values decreased the number of variables that differed significantly between the FLs to 131 (1.5%), 37 (0.4%), and 691 (7.9%) for *pinaster*,* nigra,* and *sylvestris*, respectively.

**Table 4:** Number of variables with significant differences (*P *<* *0.05) between folivory levels (FLs) for each pine species in one‐way ANOVAs (8737 variables) before and after false‐positive control with Benjamini–Hochberg tests. The percentage of differing variables relative to the total is shown in parentheses

	Number of variables with significant differences between FLs (*P *<* *0.05)	
	Without false‐positive control (%)	After Benjamini–Hochberg correction of *P* values (%)
*Pinus pinaster*	1863 (21.3)	131 (1.5)
*Pinus nigra*	1126 (12.9)	37 (0.4)
*Pinus sylvestris*	2396 (27.4)	691 (7.9)

Significant (*P *<* *0.05) and marginally significant (*P *<* *0.1) differences for each known variable are marked in Figure 5. The results of the one‐way ANOVA for the known variables are shown in Table S3. Elemental compositions and stoichiometries did not differ significantly between the FLs in *pinaster*, but *nigra* and *sylvestris* ATs had higher concentrations of K. The needles of *sylvestris* ATs also had higher concentrations of N and P. The concentrations of most sugars were lower in the needles of *pinaster* and *sylvestris* ATs but higher in *nigra*. The *pinaster* and *nigra* NATs had higher overall concentrations of amino acids than the ATs, although only a few were significantly higher. The *pinaster* NATs had higher concentrations of arginine and proline, marginally higher concentrations of glutamine and lower concentrations of leucine. The *nigra* NATs had higher concentrations of arginine, phenylalanine, and tryptophan and marginally higher concentrations of valine. The *sylvestris* NATs had higher concentrations of phenylalanine, lower concentrations of glutamic acid and serine, and marginally lower concentrations of valine. The *sylvestris* ATs had higher concentrations of adenosine and lower concentrations of uracil. The concentrations of nucleosides and purines in *pinaster* and *nigra* did not differ significantly between the FLs. The *pinaster* ATs had marginally significantly lower concentrations of succinic acid. The *nigra* ATs had higher concentrations of most of the detected organic acids associated with the Krebs cycle (citric acid, pyruvic acid, and marginally *α*‐ketoglutaric acid and fumaric acid). The *sylvestris* ATs had higher concentrations of *α*‐ketoglutaric acid and lower concentrations of succinic acid. Only a few of the detected polyphenolics differed between FLs; the *pinaster* ATs had higher concentrations of myricetin, marginally higher concentrations of vanillic acid, and marginally lower concentrations of epigallocatechin. The *nigra* ATs had marginally higher concentrations of epigallocatechin and marginally lower concentrations of taxifolin. The *sylvestris* ATs had significantly lower concentrations of myricetin. The NATs of all species had higher concentrations of terpenes, although only the concentrations of caryophyllene, sabinene, and thujone were significantly higher in *pinaster* and only the concentrations of thujone were significantly higher in *sylvestris*. None of the detected terpenes differed significantly between the FLs in *nigra*. The concentrations of D‐pinitol were significantly lower in the *pinaster* ATs and marginally higher in the *nigra* ATs. The concentrations of *δ*‐tocopherol were higher in the needles of the *nigra* and *sylvestris* but not *pinaster* ATs. The *sylvestris* ATs had lower concentrations of quinic acid and marginally lower concentrations of shikimic acid.

## Discussion

The three pine species are phylogenetically closely related but had different stoichiometries and metabolomes (Table 1), in agreement with other studies with other closely related plant species (Palama et al. 2012; Hagel et al. 2015; Rivas‐Ubach et al. 2016). *P*. *pinaster*, however, was the most divergent species (Figs. 2, 3). The *pinaster–nigra* and *pinaster–sylvestris* distances did not indicate significant metabolic differences when the complete data set was used, but the metabolomic distances calculated with the first 10 PCs of the PCA, that reduced the total variability in the first PCs, indicated a relatively shorter distance between *pinaster–nigra* than *pinaster–sylvestris* (Table 3). The NAT dendrogram indicated a similar trend suggesting that *pinaster* and *sylvestris* had the most divergent metabolomes (Fig. 3). Our metabolomic results seem thus to be related with the phylogenetic relations among the three species; *nigra* and *sylvestris* are phylogenetically closer than either is to *pinaster* (Grotkopp et al. 2004; Gernandt et al. 2005). The metabolome represents the final metabolic expression of genotypes an organism in a determined moment (Fiehn 2002), including epigenetic modifications (Rapp and Wendel 2005 in New Phytologist) and the interaction with the microbiome (Peñuelas and Terradas 2014 in Trends in Plant Science), and therefore the phenotype of an organism does not have to follow strictly its genotype. However, as mentioned, the metabolomic distances calculated among the different pine species showed clearly a link between their chemical phenotype (the metabolome) and genotype (Fig. 3 and Table 3). This result indicates that genotypes still determine the phenotypes of those species in higher proportion than any epigenetic modification or the interaction between plant and microbiome. This relationship indicates that the overall metabolomes, even with their large plasticity, are strongly constrained by evolutionary mechanisms.

The FLs differed significantly in all species (Table 2), suggesting that the systemic metabolomes of the pines were affected by PPM attack. In addition, PC2 of the case plot of the PCA separated the ATs from the NATs of the three species (although close, the separation of the FLs was not significant in *nigra*) and clearly displaced the ATs of the three species in the same direction along the axis (Fig. 2). These results suggest that the metabolomic responses to PPM attack may have similar trends in the three species. The species × FL interaction in the PERMANOVA, however, was significant, suggesting that the overall metabolomic responses to PPM attack varied with the species (Table 1). This result was supported by the additional PCA using the values of the residuals of ATs relative to NATs (Fig. 4A), by the dendrograms calculated with the residuals of the ATs relative to NATs (Fig. 4B) and by the PLS‐DA for each species separately, with FL as the discriminant factor (Fig. 5). The ATs clustered well for each species in the additional PCA of the residuals, indicating that the overall metabolomic responses to PPM attack differed among the species (Fig. 4A). In addition, the dendrogram of the residuals of the ATs identified *pinaster* as the species with the most divergent responses to PPM attack, and *nigra* and *sylvestris* were more closely represented (Fig. 4B).

The global metabolomic responses to PPM attacks differed significantly in each of the species, but the patterns to PPM attack among the species were not clear for the assigned metabolites, except for only a few common responses (Fig. 5). The most prominent trend among the three species was the higher concentrations of terpenes in the NATs than the ATs (not significant in *nigra*) (Fig. 5; Table S3). This result supports the hypothesis that rates of folivory are less intense in plants with higher concentrations of terpenes (Kessler and Baldwin 2001; Hódar et al. 2004; Achotegui‐Castells et al. 2013).

The higher concentrations of N, P, and K in *sylvestris* ATs (Fig. 5) support the hypothesis that folivores tend to select foliage with higher nutritional properties (Sterner and Elser 2002; Sardans et al. 2012). The elemental compositions of the *pinaster* and *nigra* needles, however, did not differ substantially between the FLs (Fig. 5), and only the *nigra* ATs had higher concentrations of K, thus indicating high homeostasis of foliar nutrients under PPM attack. These results likely indicate that nutrients do not play key roles in stand selection by the PPM, as recently proposed in other studies (Jactel et al. 2015; Rivas‐Ubach et al. 2016), suggesting that other physical or chemical barriers may be more important for stand selection (Tremmel and Müller 2013; Onodera et al. 2014).

The *pinaster* and *nigra* NATs, but not *sylvestris* NATs, generally had higher concentrations of most of the identified amino acids which could be related with a more activated growth‐related metabolism (Rivas‐Ubach et al. 2012) (Fig. 5). However, the metabolism of amino acids is complex because it involves a variety of intermediates from several metabolic pathways (Buchanan et al. 2015) making thus the interpretation of significant shifts of specific amino acids between FLs challenging. The concentrations of organic acids associated with the Krebs cycle differed somewhat between the FLs in *nigra* and *sylvestris*, but the lack of significant differences between the FLs in *pinaster* and the lack of differences in the foliar elemental compositions and stoichiometries were also indicators of a homeostatic central metabolism.

The synthesis of antioxidant metabolites in plants is commonly induced by folivory (Orozco‐Cardenas and Ryan 1999; Rivas‐Ubach et al. 2014, 2016). The *nigra* and *sylvestris* ATs had higher concentrations of *δ*‐tocopherol (vitamin E), but concentrations did not differ between FLs in *pinaster*, suggesting a lack of, or a minor, systemic response to oxidative stress (Fig. 5). Tocopherols are important antioxidants that protect biomembranes from reactive oxygen species (Munné‐Bosch and Peñuelas 2004; Falk and Munné‐Bosch 2010) by forming a tocopheryl radical that is ultimately reduced by hydrogen donors (Traber and Stevens 2011). Tocopherols also increased in two *P. sylvestris* subspecies under folivory by PPMs (Rivas‐Ubach et al. 2016), in accordance with our results for *sylvestris*.

Several identified phenolics have an antioxidant function (Rice‐Evans et al. 1996) and have been associated with defensive roles against folivory (Bennett and Wallsgrove 1994), but their antifeedant properties remain unclear, especially in conifers (Mumm and Hilker 2006; Rivas‐Ubach et al. 2014, 2016). Our results, independently of the role of phenolics, indicated that the three species did not generally have higher phenolic concentrations in the ATs, especially in *nigra* and *sylvestris* where none of the concentrations of the identified phenolics differed significantly from those in the NATs (Fig. 5). The clear clustering in the PCA using the residuals of the ATs relative to NATs (Fig. 4A) and the lack of clear differences in the identified metabolites between the FLs, including metabolites associated with folivory (Fig. 5), support the hypothesis that phylogenetically closely related plant species may have divergent molecular responses to herbivorous attack (Becerra 1997; Kursar et al. 2009). Nevertheless, as stated above, we detected some differences between two of the species, such as the higher concentrations of amino acids in the *pinaster* and *nigra* NATs and the higher concentrations of K and *δ*‐tocopherols in the *nigra* and *sylvestris* ATs. Furthermore, the PCAs and dendrograms for all systemic metabolomic responses to PPM attack also indicated that the response of the trees to folivory was linked to their phylogeny (Fig. 4A,B), with *pinaster* having the most divergent responses to folivory. These results suggested that metabolomic responses to folivory also carry strong phylogenetic signals in the three species, in agreement with recent studies (Carrillo‐Gavilán et al. 2015).

Hódar et al. (2002) reported lower mortality rates of first‐instar larvae feeding on *nigra* and *sylvestris* compared to *pinaster*. Moreover, *nigra* has been widely described as the staple food of PPM in most parts of Europe (Jactel et al. 2015). The metabolomic fingerprints of the three species nonetheless indicated that *nigra* had the fewest significant differences between the FLs and *sylvestris* had the most (Table 4). The *Pinus* genus has been radiating since the Mesozoic (87–193 MYA), resulting in the largest coniferous genus (Farjon 1999; Grotkopp et al. 2004; Gernandt et al. 2005; Morse et al. 2009) that now occurs in a wide global range of climates and distributions. Both *nigra* and *sylvestris* have been attacked by the PPM during the 20th century when the minimum temperatures in winter were suitable for larval survival (Torrent 1958; Démolin 1969; Huchon and Démolin 1971). However, the fact that *nigra* and *sylvestris* live in higher altitudes than *pinaster,* where typically the temperatures are lower, reduces the probability to be attacked by the caterpillars of PPM. Additionally, the viability of larvae and the intensity of defoliation depend also on the amount of predators and parasitoids that typically decrease with altitude (Hódar 2015). The distribution of *nigra* and *sylvestris* and the different responses to folivory suggest that coevolution with the PPM was probably not a determining factor driving the metabolic diversification of responses to folivory by PPM in this study. We hypothesize that the unresponsive behavior under PPM attack in *nigra*, a species with less intense and shorter coevolutionary history with the PPM compared to *pinaster*, could explain why its needles are very appetizing for the caterpillars of PPM, simply because folivores would take advantage of food sources that do not respond strongly to attack. Still, there is no scientific evidence so far and further research is warranted for a better understanding of whether a longer and more intense plant–insect interaction generally may drive to higher plant resistance to folivores and whether passivity of a plant species to folivory is definitely a significant cause of being highly attacked. Our metabolomic results, however, suggest that increases or decreases in metabolomic responses to PPM attacks, including the production of defensive compounds, should not be directly linked to the selective pressure exerted by insects on the evolutionary processes of plants. For example, both *pinaster* and *nigra* had fewer significant differences between the FLs than *sylvestris* (Table 4), but *pinaster* is typically less attractive pines for the PPMs, and *nigra* and *sylvestris* populations are now highly threatened by this lepidopteran (Jactel et al. 2015). This study thus addressed a complex scenario with multiple interactive factors and trade‐offs that produced different metabolomic responses to PPM folivory in the species. Determining whether the main metabolomic responses to the attack of PPM, including those of defensive compounds, are due to phylogeny (Carrillo‐Gavilán et al. 2015) or to the historical interaction of plant–insect coevolution (Kursar et al. 2009; Endara et al. 2015) is thus not trivial, because both hypotheses may play important roles, as supported by our results (Figs. 4A, 5).

## Conclusions

The overall metabolomes of the three pine species differed substantially. The metabolomic distances among the three species were related to their evolutionary relationships, suggesting that genotypes play stronger roles than possible epigenetic modifications in determining the chemical phenotypes of the studied pines.

- Systemic metabolomic changes in response to attacks by the caterpillars of the processionary moth differed for each species. These species‐dependent metabolomic responses to folivory were associated with the evolutionary relationships among the pines, suggesting that the plant responses to herbivory were also constrained by a strong phylogenetic component.
- Systemic phenolic metabolism did not differ importantly between the FLs in any of the species. The ATs of all species nevertheless had higher concentrations of terpenes.
- Our results suggest that both macroevolutionary history (phylogeny) and the coevolutionary history between plants and insects may both play important roles in determining the metabolomic responses of pines to PPM attack. Whether the main metabolomic responses of pines to PPM are determined by phylogeny or plant–insect coevolution is inconclusive because the response to folivory depends on the plant species and the set of trade‐offs and evolutionary factors. As our study shows, the research made on the relationship between closely related plant species with specialist folivores are excellent study cases to investigate about the phylogenetic and coevolution contribution in the metabolomic responses of plants, however, these studies will be constrained to the relatively low existence of different closely related plant species attacked by a same folivore.

## Conflict of Interest

None declared.

## Supporting information


**Table S1.** Parameters for processing LC‐MS chromatograms of the three pine species for both positive and negative ionization modes.
**Table S2.** Retention time (RT) and mass‐to‐charge ratio (*m*/*z*) of the deconvoluted ions in both negative and positive ionization modes assigned to metabolites by MZmine v.2.12.
**Table S3.** One‐way ANOVAs for each pine species of all stoichiometric variables and assigned metabolites extracted from the needles for the non‐attacked trees (NATs) and the attacked trees (ATs) for *Pinus pinaster*,* P. nigra* and *P. sylvestris*.

## References

[ece32206-bib-0001] Achotegui‐Castells, A. , J. Llusia , J. A. Hódar , and J. Peñuelas . 2013 Needle terpene concentrations and emissions of two coexisting subspecies of Scots pine attacked by the pine processionary moth (*Thaumetopoea pityocampa*). Acta Physiol. Plant 35:3047–3058.

[ece32206-bib-0002] Avtzis, N. 1986 Development of *Thaumetopoea pityocampa* Schiff. (Lepidoptera: Thaumetopoeidae) in relation to food consumption. For. Ecol. Manage. 15:65–68.

[ece32206-bib-0003] Battisti, A. 1988 Host‐plant relationships and population dynamics of the Pine Processionary Caterpillar *Thaumetopoea pityocampa* (Denis & Schiffermuller). J. Appl. Entomol. 105:393–402.

[ece32206-bib-0004] Battisti, A. , M. Stastny , S. Netherer , C. Robinet , A. Schopf , A. Roques , et al. 2005 Expansion of geographic range in the pine processionary moth caused by increased winter temperatures. Ecol. Appl. 15:2084–2096.

[ece32206-bib-0005] Battisti, A. , M. Stastny , E. Buffo , and S. Larsson . 2006 A rapid altitudinal range expansion in the pine processionary moth produced by the 2003 climatic anomaly. Glob. Change Biol. 12:662–671.

[ece32206-bib-0006] Battisti, A. , M. Avci , D. N. Avtzis , M. L. Ben Jamma , L. Berardi , W. Berretima , et al. 2015 Natural history of the processionary moths (*Thaumetopoea* spp.): new insights in relation to climate change. *Thaumetopoea pityocampa* Pp 15–80 *in* Roques A., ed. Processionary moths and climate change: an update. Springer‐Quae, Dordrecht, Netherlands.

[ece32206-bib-0007] Becerra, J. X. 1997 Insects on plants: macroevolutionary chemical trends in host use. Science 276:253–256. 9092474 10.1126/science.276.5310.253

[ece32206-bib-0008] Benigni, M. , and A. Battisti . 1999 Variazioni climatiche e processionaria del pino: adattamenti di un defoliatore a condizioni ambientali mutevoli. L'Italia Forestale e Montana 54:76–86.

[ece32206-bib-0009] Bennett, R. N. , and R. M. Wallsgrove . 1994 Secondary metabolites in plant defence mechanisms. New Phytol. 127:617–633. 10.1111/j.1469-8137.1994.tb02968.x 33874382

[ece32206-bib-0010] Blanca, G. , M. Cueto , M. J. Martínez‐Lirola , and J. Molero‐Mesa . 1998 Threatened vascular flora of Sierra Nevada (Southern Spain). Biol. Conserv. 85:269–285.

[ece32206-bib-0011] Blomberg, S. P. , T. Garland , and A. R. Ives . 2003 Testing for phylogenetic signal in comparative data: behavioral traits are more labile. Evolution 57:717–745. 12778543 10.1111/j.0014-3820.2003.tb00285.x

[ece32206-bib-0012] Buchanan, B. , W. Gruissem , K. Vickers , and R. Jones . 2015 Biochemistry and molecular biology of plants. Wiley Blackwell, Oxford, UK pp. 289–226.

[ece32206-bib-0013] Campbell, S. A. 2015 Ecological mechanisms for the coevolution of mating systems and defence. New Phytol. 205:1047–1053. 25729803 10.1111/nph.13212

[ece32206-bib-0014] Carrillo‐Gavilán, A. , X. Moreira , R. Zas , A. Gonzalez‐Voyer , M. Vilà , and L. Sampedro . 2015 Phylogenetic and biogeographical patterns in defensive strategies and quantitative allocation to chemical defences in Palaearctic and Nearctic pine trees. J. Biogeogr. 42:684–693.

[ece32206-bib-0015] Cuadros, R. , and J. R. Francia . 1999 Caracterización del sitio de ensayo de especies forestales de Lanjarón, vertiente sur de Sierra Nevada: aspectos climatológicos y fitoclimáticos. Investigación agraria. Sistemas y recursos forestales. Instituto Nacional de Investigación y Tecnología Agraria y Alimentaria (INIA) 8:143–158.

[ece32206-bib-0016] Le Cao, K. , I. Gonzalez , and S. Dejean . 2015 mixOmics: Omics Data Integration Project. R package version 5.2.0. http://CRAN.R-project.org/package=mixOmics

[ece32206-bib-0017] Démolin, G. 1969 Bioecología de la procesionaria del pino *Thaumetopoea piotyocampa* Schiff. Incidencia de los factores climáticos. Boletín del Servicio de Plagas Forestales 12:9–24.

[ece32206-bib-0018] Endara, M.‐J. , A. Weinhold , J. E. Cox , N. L. Wiggins , P. D. Coley , and T. A. Kursar . 2015 Divergent evolution in antiherbivore defences within species complexes at a single Amazonian site. J. Ecol. 103:1107–1118.

[ece32206-bib-0019] Falk, J. , and S. Munné‐Bosch . 2010 Tocochromanol functions in plants: antioxidation and beyond. J. Exp. Bot. 61:1549–1566. 20385544 10.1093/jxb/erq030

[ece32206-bib-0020] Farjon, A. 1999 World checklist and bibliography of conifers. Nord. J. Bot. 19:148.

[ece32206-bib-0021] Fiehn, O. 2002 Metabolomics – the link between genotypes and phenotypes. Plant Mol. Biol. 48:155–171. 11860207

[ece32206-bib-0022] Fox, J. , and S. Weisberg . 2011 An {R} Companion to Applied Regression. Second Edition. http://socserv.socsci.mcmaster.ca/jfox/Books/Companion

[ece32206-bib-0023] Gernandt, D. S. , G. G. López , S. O. García , and A. Liston . 2005 Phylogeny and classification of *Pinus* . Taxon 54:29–42.

[ece32206-bib-0024] Grotkopp, E. , M. Rejmánek , M. J. Sanderson , and T. L. Rost . 2004 Evolution of genome size in pines (*Pinus*) and its life‐history correlates: supertree analyses. Evolution 58:1705–1729. 15446425 10.1111/j.0014-3820.2004.tb00456.x

[ece32206-bib-0025] Hagel, J. M. , R. Mandal , B. Han , J. Han , D. R. Dinsmore , C. H. Borchers , et al. 2015 Metabolome analysis of 20 taxonomically related benzylisoquinoline alkaloid‐producing plants. BMC Plant Biol. 15:220. 26369413 10.1186/s12870-015-0594-2 PMC4570626

[ece32206-bib-0026] Heil, M. 2009 Damaged‐self recognition in plant herbivore defence. Trends Plant Sci. 14:356–363. 19540148 10.1016/j.tplants.2009.04.002

[ece32206-bib-0027] Heil, M. 2014 Herbivore‐induced plant volatiles: targets, perception and unanswered questions. New Phytol. 204:297–306.

[ece32206-bib-0028] Heil, M. , and J. C. S. Bueno . 2007 Herbivore‐induced volatiles as rapid signals in systemic plant responses: how to quickly move the information? Plant Signal. Behav. 2:191–193. 19704694 10.4161/psb.2.3.4151 PMC2634055

[ece32206-bib-0029] Herms, D. A. , and W. J. Mattson . 1992 The dilemma of plants: to grow or defend. Q. Rev. Biol. 67:283.

[ece32206-bib-0030] Hoch, G. , E. P. Toffolo , S. Netherer , A. Battisti , and A. Schopf . 2009 Survival at low temperature of larvae of the pine processionary moth *Thaumetopoea pityocampa* from an area of range expansion. Agric. For. Entomol. 11:313–320.

[ece32206-bib-0031] Hódar, J. A. 2015 Incidencia de la procesionaria del pino como consecuencia del cambio climático: previsiones y posibles soluciones Pp. 295–302 *in* Herrero A., Zavala M. A., eds. Los Bosques y la Biodiversidad frente al Cambio Climático: Impactos, Vulnerabilidad y Adaptación en España. Ministerio de Agricultura, Alimentación y Medio Ambiente, Madrid.

[ece32206-bib-0032] Hódar, J. A. , and R. Zamora . 2004 Herbivory and climatic warming: a Mediterranean outbreaking caterpillar attacks a relict, boreal pine species. Biodivers. Conserv. 13:493–500.

[ece32206-bib-0033] Hódar, J. A. , R. Zamora , and J. Castro . 2002 Host utilisation by moth and larval survival of pine processionary caterpillar *Thaumetopoea pityocampa* in relation to food quality in three *Pinus* species. Ecol. Entomol. 27:292–301.

[ece32206-bib-0034] Hódar, J. A. , J. Castro , and R. Zamora . 2003 Pine processionary caterpillar *Thaumetopoea pityocampa* as a new threat for relict Mediterranean Scots pine forests under climatic warming. Biol. Conserv. 110:123–129.

[ece32206-bib-0035] Hódar, J. A. , R. Zamora , J. Castro , and E. Baraza . 2004 Feast and famine: previous defoliation limiting survival of pine processionary caterpillar *Thaumetopoea pityocampa* in Scots pine *Pinus sylvestris* . Acta Oecol. 26:203–210.

[ece32206-bib-0036] Hódar, J. A. , R. Zamora , and L. Cayuela . 2012 Climatic change and the incidence of a forest pest in Mediterranean ecosystems: can the North Atlantic Oscillation be used as a predictor? Clim. Change. 113:699–711.

[ece32206-bib-0037] Huchon, H. , and G. Démolin . 1971 La bioécologie de la processionaire du pin. Dispersion potentielle. Dispersion actuelle. Phytoma 225:11–20.

[ece32206-bib-0038] IPCC . 2013 Summary for Policymakers *in* Stocker T. F., Qin D., Plattner G.‐K., Tignor M., Allen S. K., Boschung J., Nauels A., Xia Y., Bex V. and Midgley P. M., eds. Climate change 2013: The Physical Science Basis. Contribution of Working Group I to the Fifth Assessment Report of the Interfovernmental Panel on Climate Change.

[ece32206-bib-0039] Jactel, H. , L. Barbaro , A. Battisti , A. Bosc , M. Branco , E. Brockerhoff , et al. 2015 Insect – Tree Interactions in *Thaumetopoea pityocampa* Pp. 265–310 *in* Roques A., ed. Processionary moths and climate change: an update. Springer‐Quae, Dordrecht, Netherlands.

[ece32206-bib-0040] Jansen, J. J. , J. W. Allwood , E. Marsden‐Edwards , W. H. van der Putten , R. Goodacre , and N. M. van Dam . 2008 Metabolomic analysis of the interaction between plants and herbivores. Metabolomics 5:150–161.

[ece32206-bib-0041] Karban, R. 2011 The ecology and evolution of induced resistance against herbivores. Funct. Ecol. 25:339–347.

[ece32206-bib-0042] Kerdelhué, C. , L. Zane , M. Simonato , P. Salvato , J. Rousselet , A. Roques , et al. 2009 Quaternary history and contemporary patterns in a currently expanding species. BMC Evol. Biol. 9:220. 19732434 10.1186/1471-2148-9-220 PMC2753568

[ece32206-bib-0043] Kessler, A. , and I. T. Baldwin . 2001 Defensive function of herbivore‐induced plant volatile emissions in nature. Science 291:2141–2144. 11251117 10.1126/science.291.5511.2141

[ece32206-bib-0044] Kessler, A. , and I. T. Baldwin . 2002 Plant responses to insect herbivory: the emerging molecular analysis. Annu. Rev. Plant Biol. 53:299–328. 12221978 10.1146/annurev.arplant.53.100301.135207

[ece32206-bib-0045] t'Kind, R. , L. de Veylder , M. Storme , D. Deforce , and J. van Bocxlaer . 2008 LC‐MS metabolic profiling of *Arabidopsis thaliana* plant leaves and cell cultures: optimization of pre‐LC‐MS procedure parameters. J. Chromatogr. B 871:37–43. 10.1016/j.jchromb.2008.06.039 18617446

[ece32206-bib-0046] Kursar, T. A. , K. G. Dexter , J. Lokvam , R. T. Pennington , J. E. Richardson , M. G. Weber , et al. 2009 The evolution of antiherbivore defenses and their contribution to species coexistence in the tropical tree genus Inga. Proc. Natl Acad. Sci. USA 106:18073–18078. 19805183 10.1073/pnas.0904786106 PMC2775284

[ece32206-bib-0047] Lee, D. Y. , and O. Fiehn . 2013 Metabolomic response of Chlamydomonas reinhardtii to the inhibition of target of rapamycin (TOR) by rapamycin. J. Microbiol. Biotechnol. 23:923–931. 23727803 10.4014/jmb.1304.04057

[ece32206-bib-0048] Mari, A. , D. Lyon , L. Fragner , P. Montoro , S. Piacente , S. Wienkoop , et al. 2013 Phytochemical composition of Potentilla anserina L. analyzed by an integrative GC‐MS and LC‐MS metabolomics platform. Metabolomics 9:599–607. 23678344 10.1007/s11306-012-0473-x PMC3651535

[ece32206-bib-0049] Mirnezhad, M. , R. R. Romero‐González , K. A. Leiss , Y. H. Choi , R. Verpoorte , and P. G. L. Klinkhamer . 2010 Metabolomic analysis of host plant resistance to thrips in wild and cultivated tomatoes. Phytochem. Anal. 21:110–117. 19866459 10.1002/pca.1182

[ece32206-bib-0050] Morse, A. M. , D. G. Peterson , M. N. Islam‐Faridi , K. E. Smith , Z. Magbanua , S. A. Garcia , et al. 2009 Evolution of genome size and complexity in *Pinus* . PLoS ONE 4:e4332. 19194510 10.1371/journal.pone.0004332 PMC2633040

[ece32206-bib-0051] Mumm, R. , and M. Hilker . 2006 Direct and indirect chemical defence of pine against folivorous insects. Trends Plant Sci. 11:351–358. 16769239 10.1016/j.tplants.2006.05.007

[ece32206-bib-0052] Munné‐Bosch, S. , and J. Peñuelas . 2004 Drought‐induced oxidative stress in strawberry tree (Arbutus unedo L.) growing in Mediterranean field conditions. Plant Sci. 166:1105–1110.

[ece32206-bib-0053] Netherer, S. , and A. Schopf . 2010 Potential effects of climate change on insect herbivores in European forests—General aspects and the pine processionary moth as specific example. For. Ecol. Manage. 259:831–838.

[ece32206-bib-0054] Nuringtyas, T. R. , R. Verpoorte , P. G. L. Klinkhamer , M. M. van Oers , and K. A. Leiss . 2014 Toxicity of Pyrrolizidine Alkaloids to Spodoptera exigua Using Insect Cell Lines and Injection Bioassays. J. Chem. Ecol. 40:609–616. 24981118 10.1007/s10886-014-0459-4

[ece32206-bib-0055] Oksanen, J. , F. Guillaume‐Blanchet , R. Kindt , P. Legendre , P. Minchin , R. O'Hara , et al. 2013 vegan: Community Ecology Package. R package version 2.3‐2. http://CRAN.R-project.org/package=vegan

[ece32206-bib-0056] Onodera, H. , M. Oguro , and S. Sakai . 2014 Effects of nutrient contents and defense compounds on herbivory in reproductive organs and leaves of Iris gracilipes. Plant Ecol. 215:1025–1035.

[ece32206-bib-0057] Orozco‐Cardenas, M. , and C. A. Ryan . 1999 Hydrogen peroxide is generated systemically in plant leaves by wounding and systemin via the octadecanoid pathway. Proc. Natl Acad. Sci. 96:6553–6557. 10339626 10.1073/pnas.96.11.6553 PMC26920

[ece32206-bib-0058] Palama, T. L. , M. Grisoni , I. Fock‐Bastide , K. Jade , L. Bartet , Y. H. Choi , et al. 2012 Metabolome of Vanilla planifolia (Orchidaceae) and related species under Cymbidium mosaic virus (CymMV) infection. Plant Physiol. Biochem. 60:25–34. 22902551 10.1016/j.plaphy.2012.07.015

[ece32206-bib-0059] Peñuelas, J. , and J. Sardans . 2009 Ecological metabolomics. Chem. Ecol. 25:305–309.

[ece32206-bib-0060] Peñuelas, J. , and M. Staudt . 2010 BVOCs and global change. Trends Plant Sci. 15:133–144. 20097116 10.1016/j.tplants.2009.12.005

[ece32206-bib-0061] Peñuelas, J. , and J. Terradas . 2014 The foliar microbiome. Trends Plant Sci. 19:278–280. 24439491 10.1016/j.tplants.2013.12.007

[ece32206-bib-0062] Pierik, R. , C. L. Ballaré , and M. Dicke . 2014 Ecology of plant volatiles: taking a plant community perspective. Plant, Cell Environ. 37:1845–1853. 24689452 10.1111/pce.12330

[ece32206-bib-0063] Pluskal, T. , S. Castillo , A. Villar‐Briones , and M. Orešič . 2010 MZmine 2: modular framework for processing, visualizing, and analyzing mass spectrometry‐based molecular profile data. BMC Bioinformatics 11:395. 20650010 10.1186/1471-2105-11-395 PMC2918584

[ece32206-bib-0064] Poelman, E. H. , J. J. A. van Loon , and M. Dicke . 2008 Consequences of variation in plant defense for biodiversity at higher trophic levels. Trends Plant Sci. 13:534–541. 18774329 10.1016/j.tplants.2008.08.003

[ece32206-bib-0065] R Core Team . 2013 R: a language and environment for statistical computing. R Core Team, Vienna.

[ece32206-bib-0066] Rapp, R. A. , and J. F. Wendel . 2005 Epigenetics and plant evolution. New Phytol. 168:81–91. 16159323 10.1111/j.1469-8137.2005.01491.x

[ece32206-bib-0067] Rice‐Evans, C. A. , N. J. Miller , and G. Paganga . 1996 Structure‐antioxidant activity relationships of flavonoids and phenolic acids. Free Radic. Biol. Med. 20:933–956. 8743980 10.1016/0891-5849(95)02227-9

[ece32206-bib-0068] Rivas‐Ubach, A. , J. Sardans , M. Pérez‐Trujillo , M. Estiarte , and J. Peñuelas . 2012 Strong relationship between elemental stoichiometry and metabolome in plants. Proc. Natl Acad. Sci. 109:4181–4186. 22371578 10.1073/pnas.1116092109 PMC3306711

[ece32206-bib-0069] Rivas‐Ubach, A. , M. Pérez‐Trujillo , J. Sardans , A. Gargallo‐Garriga , T. Parella , and J. Peñuelas . 2013 Ecometabolomics: optimized NMR‐based method. Methods Ecol. Evol. 4:464–473.

[ece32206-bib-0070] Rivas‐Ubach, A. , A. Gargallo‐Garriga , J. Sardans , M. Oravec , L. Mateu‐Castell , M. Pérez‐Trujillo , et al. 2014 Drought enhances folivory by shifting foliar metabolomes in *Quercus ilex* trees. New Phytol. 202:874–885. 24443979 10.1111/nph.12687

[ece32206-bib-0071] Rivas‐Ubach, A. , J. Sardans , J. A. Hódar , J. Garcia‐Porta , A. Guenther , M. Oravec , et al. 2016 Similar local but different systemic metabolomic responses of closely related pine subspecies to folivory by caterpillars of the processionary moth. Plant Biol. 18:484–494. 26642818 10.1111/plb.12422

[ece32206-bib-0072] Rousselet, J. , R. Zhao , D. Argal , M. Simonato , A. Battisti , A. Roques , et al. 2010 The role of topography in structuring the demographic history of the pine processionary moth, *Thaumetopoea pityocampa* (Lepidoptera: Notodontidae). J. Biogeogr. 37:1478–1490.

[ece32206-bib-0073] Sardans, J. , F. Montes , and J. Peñuelas . 2010 Determination of As, Cd, Cu, Hg and Pb in biological samples by modern electrothermal atomic absorption spectrometry. Spectrochim. Acta, Part B 65:97–112.

[ece32206-bib-0074] Sardans, J. , J. Peñuelas , and A. Rivas‐Ubach . 2011 Ecological metabolomics: overview of current developments and future challenges. Chemoecology 21:191–225.

[ece32206-bib-0075] Sardans, J. , A. Rivas‐Ubach , and J. Peñuelas . 2012 The elemental stoichiometry of aquatic and terrestrial ecosystems and its relationships with organismic lifestyle and ecosystem structure and function: a review and perspectives. Biogeochemistry 111:1–39.

[ece32206-bib-0076] Shulaev, V. , D. Cortes , G. Miller , and R. Mittler . 2008 Metabolomics for plant stress response. Physiol. Plant. 132:199–208. 18251861 10.1111/j.1399-3054.2007.01025.x

[ece32206-bib-0077] Sterner, R. , and J. Elser . 2002 Ecological stoichiometry: the biology of elements from molecules to the biosphere. Princetion University Press, Princeton, NJ, USA Pp. 1–464.

[ece32206-bib-0078] Sticher, L. , B. Mauch‐Mani , and J. P. Métraux . 1997 Systemic acquired resistance. Annu. Rev. Phytopathol. 35:235–270. 15012523 10.1146/annurev.phyto.35.1.235

[ece32206-bib-0079] Sumner, L. W. , A. Amberg , D. Barrett , M. H. Beale , R. Beger , C. A. Daykin , et al. 2007 Proposed minimum reporting standards for chemical analysis Chemical Analysis Working Group (CAWG) Metabolomics Standards Initiative (MSI). Metabolomics 3:211–221. 24039616 10.1007/s11306-007-0082-2 PMC3772505

[ece32206-bib-0080] Thompson, J. N. , and B. M. Cunningham . 2002 Geographic structure and dynamics of coevolutionary selection. Nature 417:735–738. 12066183 10.1038/nature00810

[ece32206-bib-0081] Torrent, J. A. 1958 Tratamientos de la procesionaria del pino (*Thaumetopoea pityocampa* Schiff.). Boletín del Servicio de Plagas Forestales 2:65–80.

[ece32206-bib-0082] Traber, M. G. , and J. F. Stevens . 2011 Vitamins C and E: beneficial effects from a mechanistic perspective. Free Radic. Biol. Med. 51:1000–1013. 21664268 10.1016/j.freeradbiomed.2011.05.017 PMC3156342

[ece32206-bib-0083] Tremmel, M. , and C. Müller . 2013 The consequences of alternating diet on performance and food preferences of a specialist leaf beetle. J. Insect Physiol. 59:840–847. 23727303 10.1016/j.jinsphys.2013.05.009

[ece32206-bib-0084] Whitham, T. G. , J. K. Bailey , J. A. Schweitzer , S. M. Shuster , R. K. Bangert , C. J. LeRoy , et al. 2006 A framework for community and ecosystem genetics: from genes to ecosystems. Nat. Rev. Genet. 7:510–523. 16778835 10.1038/nrg1877

