# Supplementary material for: Are the metabolomic responses to folivory of closely related plant species linked to macroevolutionary and plant–folivore coevolutionary processes?
Source: Ecol Evol. 2016 Jun 2;6(13):4372–86. doi: 10.1002/ece3.2206 (PMC4893459; doi:10.1002/ece3.2206)
Supplement: Supplementary file 1 — Table S1. Parameters for processing LC‐MS chromatograms of the three pine species for both positive and negative ionization modes. Table S2. Retention time (RT) and mass‐to‐charge ratio (m/z) of the deconvoluted ions in both negative and positive ionization modes assigned to metabolites by MZmine v.2.12. Table S3. One‐way ANOVAs for each pine species of all stoichiometric variables and assigned metabolites extracted from the needles for the non‐attacked trees (NATs) and the attacked trees (ATs) for Pinus pinaster, P. nigra and P. sylvestris. [file ECE3-6-4372-s001.docx]

**Supporting information.**

**Rivas-Ubach et al., 2016**

**Table S1. Parameters for processing** LC-MS chromatograms of the three pine species for both positive and negative ionization modes. Chromatograms were processed by MZmine 2.12.

|  |  | (+H) Chromatograms |  | (-H) Chromatograms |
| --- | --- | --- | --- | --- |
| **1** | **Baseline correction –**  **RollingBall baseline corrector** |  |  |  |
|  | Width of local window for minimization/maximization (wm) | 10 |  | 12 |
|  | Width of local window for smoothing (ws) | 8 |  | 10 |
| **2** | **Mass detection - Exact Mass** |  |  |  |
|  | Noise level | 1.0 × 10^5^ |  | 3.0 × 10^4^ |
| **3** | **Chromatogram builder** |  |  |  |
|  | Minimum time span | 0.05 |  | 0.05 |
|  | Minimum height | 1.0 × 10^4^ |  | 3.0 × 10^4^ |
|  | m/z tolerance | 0.0005 |  | 0.0005 |
| **4** | **Smoothing** |  |  |  |
|  | Filter width | 5 |  | 5 |
| **5** | **Chromatogram deconvolution –**  **Local minimum search** |  |  |  |
|  | Chromatographic threshold | 30% |  | 30% |
|  | Search minimum in RT range (min) | 0.1 |  | 0.1 |
|  | Minimum relative height | 5.0% |  | 5.0% |
|  | Minimum absolute height | 1.0 × 10^4^ |  | 1.0 × 10^4^ |
|  | Minimum ratio of peak top/edge | 2 |  | 2 |
|  | Peak duration range | 0.0-0.5 |  | 0.0-0.5 |
| **6** | **Chromatogram alignment (join alignment)** |  |  |  |
|  | m/z tolerance | 0.0005 |  | 0.0005 |
|  | Weight for m/z | 80 |  | 80 |
|  | RT tolerance | 0.2 |  | 0.2 |
|  | Weight for RT | 20 |  | 20 |
| **7** | **Gap filling (Peak Finder)** |  |  |  |
|  | Intensity tolerance | 30% |  | 30% |
|  | m/z tolerance | 0.0005 |  | 0.0005 |
|  | Retention-time tolerance | 0.3 |  | 0.2 |
|  | RT correction | marked |  | marked |
| **8** | **Metabolite Assignation** |  |  |  |
|  | m/z tolerance | 0.0005 |  | 0.0005 |
|  | RT tolerance | 0.3 |  | 0.3 |

**Table S2.** Retention time (RT) and mass-to-charge ratio (m/z) of the deconvoluted ions in both negative and positive ionization modes assigned to metabolites by MZmine v.2.12. The assignment of the metabolites was based on the standards. RT, m/z and peak range of the standards are shown in the table. The errors of m/z and RT of the ions assigned to metabolites relative to the errors of m/z and RT of the standards are shown.

|  | | **Experimental** | | **Standard** | | **Assignation error,**  **Experimental vs. Standard** | | | |
| --- | --- | --- | --- | --- | --- | --- | --- | --- | --- |
| **Ionization mode** | **Molecular compound** | **row m/z** | **RT** | **m/z** | **RT** | **m/z error** | **RT error** | | |
| (-) | Dihydroxytrimethoxyflavone (Flav) | 343.08216 | 16.67 | 343.08252 | 16.42 | 0.00044 | 0.25 | | |
| (-) | a.ketoglutaric acid | 145.01452 | 1.50 | 145.01483 | 1.65 | -0.00056 | -0.15 | | |
| (-) | Abscisic acid (ABA) | 263.12877 | 13.57 | 263.12888 | 13.56 | -0.00036 | 0.01 | | |
| (-) | Catechin | 289.07228 | 3.42 | 289.07254 | 3.39 | -0.00031 | 0.03 | | |
| (-) | Catechin | 289.07230 | 3.41 | 289.07254 | 3.39 | -0.00011 | 0.02 | | |
| (-) | Chlorogenic acid | 353.08751 | 3.11 | 353.08771 | 3.02 | 0.00025 | 0.09 | | |
| (-) | Citric acid | 173.00949 | 1.51 | 173.00925 | 1.77 | 0.00026 | -0.26 | | |
| (-) | Citric acid | 173.00954 | 1.51 | 173.00925 | 1.77 | 0.00000 | -0.26 | | |
| (-) | Citric acid | 191.02012 | 1.50 | 191.01976 | 1.77 | 0.00009 | -0.27 | | |
| (-) | Deoxyglucose - Deoxygalactose - D-Fucose | 163.06135 | 1.48 | 163.06145 | 1.45 | -0.00010 | 0.03 | | |
| (-) | Disaccharides | 341.10786 | 1.46 | 341.10776 | 1.42 | -0.00021 | 0.04 | | |
| (-) | D-pinitol | 193.07137 | 1.52 | 193.07152 | 1.38 | -0.00011 | 0.14 | | |
| (-) | δ-tocopherol | 401.12884 | 1.43 | 401.12906 | 1.35 | -0.00034 | 0.08 | | |
| (-) | Epicatechin | 289.07223 | 5.19 | 289.07254 | 5.33 | -0.00024 | -0.14 | | |
| (-) | Epicatechin | 289.07224 | 5.18 | 289.07254 | 5.33 | -0.00044 | -0.15 | | |
| (-) | Epicatechin | 289.07214 | 5.19 | 289.07254 | 5.33 | -0.00045 | -0.14 | | |
| (-) | Epigallocatechin | 305.06738 | 2.61 | 305.06772 | 2.64 | -0.00026 | -0.03 | | |
| (-) | Ferulic acid | 193.05066 | 10.51 | 193.05095 | 10.59 | -0.00024 | -0.08 | | |
| (-) | Fumaric acid | 115.00399 | 1.49 | 115.00389 | 1.7 | -0.00020 | -0.21 | | |
| (-) | Fumaric acid | 115.00400 | 1.48 | 115.00389 | 1.7 | 0.00003 | -0.22 | | |
| (-) | Gibberellic acid (GA3) | 345.13399 | 10.93 | 345.13391 | 10.63 | 0.00024 | 0.30 | | |
| (-) | Gibberellic acid (GA3) | 345.13416 | 10.44 | 345.13391 | 10.63 | 0.00029 | -0.19 | | |
| (-) | Hexoses | 143.03526 | 1.49 | 143.03496 | 1.43 | 0.00036 | 0.06 | | |
| (-) | Hexoses | 179.05632 | 1.46 | 179.05595 | 1.43 | -0.00010 | 0.03 | | |
| (-) | Hexoses | 179.05632 | 1.46 | 179.05595 | 1.43 | 0.00010 | 0.03 | | |
| (-) | Kaempferol | 285.04025 | 14.82 | 285.04059 | 14.81 | -0.00015 | 0.01 | | |
| (-) | Luteolin | 285.04030 | 13.63 | 285.04059 | 13.68 | -0.00022 | -0.05 | | |
| (-) | Myricetin | 317.03075 | 12.46 | 317.03091 | 12.47 | -0.00031 | -0.01 | | |
| (-) | Myricetin | 317.03082 | 12.46 | 317.03091 | 12.47 | -0.00030 | -0.01 | | |
| (-) | Pantothenic acid (Vit B5) | 218.10312 | 2.00 | 218.10324 | 2.01 | -0.00040 | -0.01 | | |
| (-) | Pentoses | 149.04574 | 1.48 | 149.04564 | 1.43 | -0.00034 | 0.05 | | |
| (-) | Pentoses | 149.04583 | 1.48 | 149.04564 | 1.43 | -0.00029 | 0.05 | | |
| (-) | Protocatechuic acid | 153.01943 | 2.57 | 153.01987 | 2.59 | 0.00010 | -0.02 | | |
| (-) | Pyruvic acid | 87.00899 | 1.52 | 87.00888 | 1.65 | 0.00011 | -0.13 | | |
| (-) | Pyruvic acid | 87.00907 | 1.48 | 87.00888 | 1.65 | -0.00054 | -0.17 | | |
| (-) | Quercetin | 301.03546 | 13.73 | 301.03598 | 13.73 | 0.00008 | 0.00 | | |
| (-) | Quinic acid | 191.05598 | 1.51 | 191.05588 | 1.47 | 0.00025 | 0.04 | | |
| (-) | Quinic acid | 191.05611 | 1.43 | 191.05588 | 1.47 | -0.00011 | -0.04 | | |
| (-) | Shikimic acid | 173.04551 | 1.62 | 173.04553 | 1.63 | 0.00004 | -0.01 | | |
| (-) | Shikimic acid | 173.04590 | 1.61 | 173.04553 | 1.63 | 0.00051 | -0.02 | | |
| (-) | Shikimic acid | 173.04582 | 1.63 | 173.04553 | 1.63 | 0.00052 | 0.00 |  |  |
| (-) | Sodium salicylate | 137.02458 | 10.46 | 137.02457 | 10.51 | -0.00009 | -0.05 | | |
| (-) | Succinic acid | 117.01948 | 1.53 | 117.01937 | 1.74 | -0.00006 | -0.21 | | |
| (-) | Taxifolin | 303.05090 | 11.05 | 303.05127 | 11.09 | 0.00030 | -0.04 | | |
| (-) | Tryptophan | 203.08249 | 2.47 | 203.08261 | 2.36 | 0.00037 | 0.11 | | |
| (-) | Tryptophan | 203.08249 | 2.47 | 203.08261 | 2.36 | 0.00037 | 0.11 | | |
| (-) | Vanillic acid | 167.03532 | 4.61 | 167.03564 | 4.67 | -0.00004 | -0.06 | | |
| (-) | Xylitol - Arabitol | 151.06142 | 1.15 | 151.06154 | 1.4 | -0.00003 | -0.25 | | |
| (+) | Dihydroxytrimethoxyflavone (Flav) | 345.09639 | 16.68 | 345.09595 | 16.39 | -0.00022 | 0.29 | |  |
| (+) | Dihydroxytrimethoxyflavone (Flav) | 345.09661 | 16.67 | 345.09717 | 16.39 | -0.00021 | 0.28 | | |
| (+) | Adenine | 136.06150 | 1.68 | 136.06125 | 1.77 | -0.00034 | -0.09 | | |
| (+) | Adenine | 136.06151 | 1.60 | 136.06125 | 1.77 | -0.00038 | -0.17 | | |
| (+) | Adenosine | 268.10364 | 1.63 | 268.10364 | 1.75 | -0.00029 | -0.12 | | |
| (+) | Adenosine | 268.10373 | 1.71 | 268.10364 | 1.75 | -0.00015 | -0.04 | | |
| (+) | Alanine | 90.05450 | 1.52 | 90.0546 | 1.43 | -0.00013 | 0.09 | | |
| (+) | Arginine | 175.11879 | 1.41 | 175.1190 | 1.34 | -0.00011 | 0.07 | | |
| (+) | Arginine | 175.11889 | 1.41 | 175.1190 | 1.34 | -0.00016 | 0.07 | | |
| (+) | Caryophyllene | 203.17936 | 21.47 | 203.1797 | 21.46 | -0.00009 | 0.01 | | |
| (+) | Caryophyllene | 221.18976 | 21.48 | 221.19 | 21.46 | 0.00008 | 0.02 | | |
| (+) | Catechin | 291.08678 | 3.44 | 291.08722 | 3.42 | -0.00012 | 0.02 | | |
| (+) | Catechin | 291.08677 | 3.40 | 291.08722 | 3.42 | 0.00010 | -0.02 | | |
| (+) | Choline | 104.10661 | 1.47 | 104.10658 | 1.31 | 0.00019 | 0.16 | | |
| (+) | Gibberellic acid (GA1) | 349.16331 | 10.50 | 349.16385 | 10.61 | 0.00038 | -0.11 | | |
| (+) | Glutamic acid | 148.06024 | 1.58 | 148.06035 | 1.43 | -0.00038 | 0.15 | | |
| (+) | Glutamic acid | 148.06039 | 1.52 | 148.06035 | 1.43 | -0.00024 | 0.09 | | |
| (+) | Glutamine | 130.04951 | 1.67 | 130.0490 | 1.46 | -0.00015 | 0.21 | | |
| (+) | Glutamine | 130.04952 | 1.61 | 130.0490 | 1.46 | -0.00007 | 0.15 | | |
| (+) | Glutamine | 147.07621 | 1.52 | 147.0763 | 1.46 | -0.00003 | 0.06 | | |
| (+) | Glutamine | 147.07624 | 1.52 | 147.0763 | 1.46 | -0.00044 | 0.06 | | |
| (+) | Isoleucine | 86.09596 | 1.77 | 86.0960 | 1.7 | 0.00011 | 0.07 | | |
| (+) | Isoleucine | 86.09597 | 1.81 | 86.0960 | 1.7 | 0.00019 | 0.11 | | |
| (+) | Isoleucine | 132.10168 | 1.81 | 132.1019 | 1.7 | -0.00052 | 0.11 | | |
| (+) | Isoleucine | 132.10169 | 1.60 | 132.1019 | 1.7 | 0.00010 | -0.10 | | |
| (+) | Leucine | 132.10122 | 1.51 | 132.1016 | 1.76 | 0.00023 | -0.25 | | |
| (+) | Methionine | 150.05785 | 1.36 | 150.0580 | 1.59 | 0.00020 | -0.23 | | |
| (+) | Methionine | 150.05787 | 1.32 | 150.0580 | 1.59 | -0.00009 | -0.27 | | |
| (+) | Methionine | 150.05789 | 1.33 | 150.0580 | 1.59 | -0.00009 | -0.26 | | |
| (+) | Pantothenic acid (Vit B5) | 220.11777 | 2.02 | 220.11769 | 2 | -0.00002 | 0.02 | | |
| (+) | Phenylalanine | 120.08038 | 1.93 | 120.0800 | 1.91 | 0.00037 | 0.02 | | |
| (+) | Phenylalanine | 166.08602 | 1.70 | 166.0864 | 1.91 | 0.00029 | -0.21 | | |
| (+) | Phenylalanine | 166.08616 | 1.93 | 166.0864 | 1.91 | 0.00001 | 0.02 | | |
| (+) | Phenylalanine | 166.08625 | 1.93 | 166.0864 | 1.91 | 0.00011 | 0.02 | | |
| (+) | Proline | 116.07023 | 1.53 | 116.0703 | 1.49 | -0.00037 | 0.04 | | |
| (+) | Proline | 116.07027 | 1.51 | 116.0703 | 1.49 | -0.00022 | 0.02 | | |
| (+) | Sabinene | 81.06940 | 21.58 | 81.0692 | 21.8 | -0.00016 | -0.22 | | |
| (+) | Sabinene | 137.13191 | 22.05 | 137.132 | 21.8 | 0.00012 | 0.25 | | |
| (+) | Serine | 106.04941 | 1.53 | 106.0495 | 1.47 | 0.00026 | 0.06 | | |
| (+) | Threonine | 102.05448 | 1.73 | 102.0547 | 1.43 | -0.00027 | 0.30 | | |
| (+) | Threonine | 102.05454 | 1.61 | 102.0547 | 1.43 | 0.00036 | 0.18 | | |
| (+) | Threonine | 120.06512 | 1.51 | 120.0650 | 1.43 | -0.00012 | 0.08 | | |
| (+) | Thujone | 153.12709 | 16.56 | 153.12683 | 16.3 | -0.00012 | 0.26 | | |
| (+) | Tryptophan | 188.07053 | 2.51 | 188.0708 | 2.49 | -0.00058 | 0.02 | | |
| (+) | Tryptophan | 205.09736 | 2.51 | 205.0970 | 2.49 | -0.00048 | 0.02 | | |
| (+) | Tyrosine | 182.08082 | 1.64 | 182.0814 | 1.77 | -0.00003 | -0.13 | | |
| (+) | Tyrosine | 182.08092 | 1.70 | 182.0814 | 1.77 | 0.00018 | -0.07 | | |
| (+) | Uracil | 113.03404 | 1.73 | 113.03407 | 1.49 | -0.00015 | 0.24 | | |
| (+) | Uridine | 245.07687 | 1.72 | 245.07669 | 1.76 | -0.00007 | -0.04 | | |
| (+) | Valine | 72.08025 | 1.45 | 72.0804 | 1.53 | 0.00003 | -0.08 | | |
| (+) | Valine | 72.08033 | 1.43 | 72.0804 | 1.53 | -0.00023 | -0.10 | | |
| (+) | Valine | 72.08043 | 1.43 | 72.0804 | 1.53 | -0.00014 | -0.10 | | |
| (+) | Valine | 118.08587 | 1.57 | 118.0861 | 1.53 | -0.00032 | 0.04 | | |
| (+) | Valine | 118.08596 | 1.52 | 118.0861 | 1.53 | -0.00012 | -0.01 | | |

**Table S3.** One-way ANOVAs for each pine species of all stoichiometric variables and assigned metabolites extracted from the needles for the non-attacked trees (NATs) and the attacked trees (ATs) for *Pinus pinaster*, *P. nigra* and *P. sylvestris*. The means for the elements are represented by percentages of the weight. The means of the assigned metabolites represent the integral values of the deconvoluted ion chromatograms.

| ***P. pinaster*** | | | | |
| --- | --- | --- | --- | --- |
|  | **NATs**  **Mean (SE)** | **ATs**  **Mean (SE)** | ***F*** | ***P*** |
| C (% wt.) | 48.6 (0.3) | 48.7 (0.1) | 0.16 | 0.68948 |
| N (% wt.) | 0.85 (0.03) | 0.82 (0.03) | 0.38 | 0.54399 |
| P (% wt.) | 0.08 (0) | 0.07 (0) | 2.85 | 0.10883 |
| K (% wt.) | 0.74 (0.05) | 0.65 (0.05) | 1.67 | 0.21303 |
| C:N | 57.9 (1.9) | 59.9 (2.2) | 0.48 | 0.49743 |
| N:P | 11 (0.7) | 12.5 (0.9) | 1.95 | 0.17991 |
| C:P | 634 (43) | 740 (46) | 2.83 | 0.10969 |
| N:K | 1.21 (0.12) | 1.34 (0.11) | 0.68 | 0.41978 |
| K:P | 9.5 (0.8) | 9.6 (0.7) | 0.02 | 0.90248 |
| Disaccharides | 409421 (61457) | 372695 (42429) | 0.24 | 0.62882 |
| Hexoses | 14604324 (1859208) | 14022948 (1909601) | 0.05 | 0.82978 |
| Pentoses | 2314834 (395461) | 1553626 (154671) | 3.21 | 0.08985 |
| Deoxyglucose - Deoxygalactose - D-Fucose (S1) | 144287 (41435) | 149444 (33532) | 0.01 | 0.92400 |
| Xylitol – Arabitol (S2) | 8539876 (690894) | 6941918 (517338) | 3.43 | 0.08059 |
| Arginine | 1806031 (516753) | 315802 (82526) | 8.11 | 0.01068 |
| Glutamine | 49084019 (7122753) | 32981060 (4434410) | 3.68 | 0.07095 |
| Isoleucine | 45891930 (9909865) | 49726783 (13992945) | 0.05 | 0.82555 |
| Leucine | 108315 (19627) | 240454 (70812) | 3.23 | 0.08893 |
| Methionine | 47122368 (7645460) | 34782432 (5061938) | 1.81 | 0.19508 |
| Phenylalanine | 66536441 (16508171) | 45121463 (10537081) | 1.20 | 0.28861 |
| Proline | 9245626 (1698737) | 4197643 (999627) | 6.56 | 0.01964 |
| Serine | 2803425 (650984) | 1634269 (330738) | 2.56 | 0.12674 |
| Threonine | 6448395 (1026918) | 5515336 (522543) | 0.66 | 0.42864 |
| Tryptophan | 239190020 (59823762) | 177506007 (40719818) | 0.73 | 0.40520 |
| Tyrosine | 2779307 (359021) | 3174567 (790359) | 0.21 | 0.65432 |
| Valine | 12734589 (1679522) | 12565845 (1282589) | 0.01 | 0.93724 |
| Adenine | 10292583 (940042) | 8511524 (1481819) | 1.07 | 0.31435 |
| Adenosine | 27391 (5519) | 86866 (32666) | 3.58 | 0.07564 |
| Uracil | 16610871 (1796072) | 16308918 (1498541) | 0.02 | 0.89872 |
| Uridine | 282164 (37790) | 212562 (38437) | 1.67 | 0.21296 |
| Α-ketoglutaric acid | 55385 (12955) | 41000 (8209) | 0.88 | 0.36067 |
| Citric acid | 16468865 (1942833) | 16020194 (746095) | 0.05 | 0.83173 |
| Succinic acid | 169683 (29761) | 104516 (12150) | 4.11 | 0.05771 |
| Pyruvic acid | 5319554 (471396) | 4528429 (320697) | 1.93 | 0.18220 |
| Dihydroxytrimethoxyflavone (Flav) | 6130265 (1552975) | 3304818 (1030960) | 2.30 | 0.14694 |
| Catechin | 66039 (7439) | 44699 (13893) | 1.83 | 0.19245 |
| Chlorogenic acid | 2506566 (217324) | 2429233 (314187) | 0.04 | 0.84185 |
| D-pinitol | 1702080 (77383) | 1197792 (142809) | 9.64 | 0.00612 |
| Epigallocatechin | 87352 (16957) | 51196 (10904) | 3.22 | 0.08971 |
| Fumaric acid | 3640189 (466224) | 3704184 (303069) | 0.01 | 0.90965 |
| Kaempferol | 595548 (195007) | 581456 (108775) | 0.00 | 0.95037 |
| Luteolin | 24305 (3690) | 19947 (4736) | 0.53 | 0.47726 |
| Myricetin | 1786823 (383052) | 5269946 (1348764) | 6.17 | 0.02305 |
| Protocatechuic acid | 112043 (22332) | 91106 (14607) | 0.62 | 0.44290 |
| Quercetin | 962703 (238427) | 1221033 (171673) | 0.77 | 0.39084 |
| Quinic acid | 2937563051 (309387880) | 3355063414 (249115694) | 1.10 | 0.30713 |
| Sodium salicylate | 381546 (86968) | 601157 (110919) | 2.43 | 0.13662 |
| Taxifolin | 347338 (92656) | 362779 (51316) | 0.02 | 0.88572 |
| Vanillic acid | 104224 (15953) | 394713 (144614) | 3.99 | 0.06122 |
| Caryophyllene | 290885 (39673) | 167101 (24706) | 7.01 | 0.01634 |
| Sabinene | 1248317 (215866) | 565246 (145271) | 6.89 | 0.01716 |
| Thujone | 2391604 (955194) | 263679 (74933) | 4.93 | 0.03943 |
| Abscisic acid (ABA) | 156013 (24628) | 130425 (22297) | 0.59 | 0.45116 |
| Choline | 10682651 (2326797) | 7274240 (1034933) | 1.79 | 0.19742 |
| δ-tocopherol | 511356 (100791) | 412385 (65303) | 0.68 | 0.42067 |
| Gibberellic acid (GA3) | 2965577 (225856) | 2774100 (343308) | 0.22 | 0.64684 |
| Pantothenic acid (Vit B5) | 3154192 (626718) | 1988904 (702774) | 1.53 | 0.23179 |
| Shikimic acid | 26440960 (3195348) | 20945830 (2752556) | 1.70 | 0.20901 |
|  | | | | |
| ***P. nigra*** | | | | |
|  | **NATs**  **Mean (SE)** | **ATs**  **Mean (SE)** | ***F*** | ***P*** |
| C (% wt.) | 49.5 (0.3) | 49.2 (0.1) | 0.93 | 0.34837 |
| N (% wt.) | 0.95 (0.13) | 0.81 (0.03) | 1.26 | 0.27680 |
| P (% wt.) | 0.09 (0) | 0.1 (0) | 1.71 | 0.20702 |
| K (% wt.) | 0.68 (0.03) | 0.81 (0.04) | 6.19 | 0.02292 |
| C:N | 56.8 (4.3) | 61.9 (2.3) | 1.10 | 0.30799 |
| N:P | 10.1 (1.1) | 8.1 (0.5) | 2.70 | 0.11800 |
| C:P | 539 (22) | 494 (22) | 2.05 | 0.16933 |
| N:K | 1.41 (0.17) | 1.03 (0.08) | 4.37 | 0.05115 |
| K:P | 7.32 (0.34) | 8.03 (0.4) | 1.83 | 0.19314 |
| Disaccharides | 427360 (27574) | 582109 (45927) | 8.35 | 0.00978 |
| Hexoses | 9082244 (1550678) | 7582568 (1031279) | 0.65 | 0.43117 |
| Pentoses | 2516296 (156024) | 3988034 (425339) | 10.55 | 0.00446 |
| Deoxyglucose - Deoxygalactose - D-Fucose (S1) | 206989 (28237) | 341289 (46270) | 6.14 | 0.02337 |
| Xylitol – Arabitol (S2) | 4500248 (220144) | 4608872 (382792) | 0.06 | 0.80847 |
| Arginine | 354512 (131735) | 51396 (17331) | 5.20 | 0.03492 |
| Glutamine | 17582309 (2381410) | 14205571 (1483345) | 1.45 | 0.24435 |
| Isoleucine | 66371147 (6710206) | 52472645 (8928352) | 1.55 | 0.22931 |
| Leucine | 214769 (46130) | 205007 (59244) | 0.02 | 0.89800 |
| Methionine | 37193886 (1947230) | 35313979 (4140884) | 0.17 | 0.68605 |
| Phenylalanine | 110276812 (16781230) | 54680900 (12116373) | 7.21 | 0.01509 |
| Proline | 4186671 (1001474) | 2868216 (577378) | 1.30 | 0.26901 |
| Serine | 1253882 (279893) | 801236 (128275) | 2.16 | 0.15878 |
| Threonine | 4429211 (624014) | 5818314 (721179) | 2.12 | 0.16245 |
| Tryptophan | 181111719 (19161060) | 107217771 (19371098) | 7.36 | 0.01428 |
| Tyrosine | 6847785 (797066) | 6221387 (635228) | 0.38 | 0.54652 |
| Valine | 5818195 (1271442) | 3393770 (532656) | 3.09 | 0.09561 |
| Adenine | 12400095 (1007657) | 9769734 (1289449) | 2.58 | 0.12538 |
| Adenosine | 1558064 (680249) | 1020887 (382642) | 0.47 | 0.50006 |
| Uracil | 10484966 (628216) | 12036463 (1250926) | 1.23 | 0.28230 |
| Uridine | 1289401 (200546) | 889891 (95938) | 3.23 | 0.08913 |
| α-ketoglutaric acid | 38234 (7992) | 61686 (9001) | 3.80 | 0.06715 |
| Citric acid | 20859046 (3044531) | 32199748 (3222865) | 6.54 | 0.01977 |
| Succinic acid | 130845 (28733) | 171411 (36382) | 0.77 | 0.39308 |
| Pyruvic acid | 3842385 (439131) | 5227080 (464462) | 4.69 | 0.04395 |
| Dihydroxytrimethoxyflavone (Flav) | 50076382 (6088372) | 42597085 (7154076) | 0.63 | 0.43631 |
| Catechin | 1947396 (618578) | 1174237 (275579) | 1.30 | 0.26853 |
| Chlorogenic acid | 644088 (276191) | 712817 (192728) | 0.04 | 0.84059 |
| D-pinitol | 1209885 (90167) | 1700937 (261010) | 3.16 | 0.09226 |
| Epigallocatechin | 560440 (79839) | 923180 (171468) | 3.68 | 0.07114 |
| Fumaric acid | 2128024 (143630) | 2714482 (240460) | 4.38 | 0.05070 |
| Kaempferol | 775401 (113918) | 570391 (153987) | 1.15 | 0.29863 |
| Luteolin | 15104 (2354) | 18201 (4206) | 0.41 | 0.52851 |
| Myricetin | 19668 (6157) | 27706 (5274) | 0.98 | 0.33456 |
| Protocatechuic acid | 137617 (29367) | 145774 (23778) | 0.05 | 0.83151 |
| Quercetin | 269859 (57729) | 332453 (76326) | 0.43 | 0.52134 |
| Quinic acid | 1869061028 (179577563) | 2049109295 (124067694) | 0.68 | 0.42023 |
| Sodium salicylate | 680357 (83173) | 570625 (59755) | 1.15 | 0.29811 |
| Taxifolin | 1552800 (305590) | 873207 (178371) | 3.69 | 0.07076 |
| Vanillic acid | 120582 (26811) | 85520 (23674) | 0.96 | 0.33993 |
| Caryophyllene | 1808836 (423457) | 2039421 (548876) | 0.11 | 0.74326 |
| Sabinene | 559204 (77563) | 624285 (192845) | 0.10 | 0.75780 |
| Thujone | 4090296 (1810130) | 1498481 (586582) | 1.86 | 0.18997 |
| Abscisic acid (ABA) | 1458272 (265078) | 1456422 (206018) | 0.00 | 0.99566 |
| Choline | 9735652 (1133122) | 7497201 (1139652) | 1.94 | 0.18063 |
| δ-tocopherol | 674507 (44933) | 1069151 (116049) | 10.06 | 0.00529 |
| Gibberellic acid (GA3) | 3395891 (228197) | 3280989 (125472) | 0.19 | 0.66431 |
| Pantothenic acid (Vit B5) | 1772832 (440952) | 2935581 (529572) | 2.85 | 0.10880 |
| Shikimic acid | 25414553 (3654029) | 29776068 (3881663) | 0.67 | 0.42397 |
|  | | | | |
| ***P. sylvestris*** | | | | |
|  | **NATs**  **Mean (SE)** | **ATs**  **Mean (SE)** | ***F*** | ***P*** |
| C (% wt.) | 48.7 (0.3) | 48.6 (0.1) | 0.09 | 0.76694 |
| N (% wt.) | 0.92 (0.03) | 1.03 (0.03) | 7.06 | 0.01605 |
| P (% wt.) | 0.11 (0) | 0.12 (0.01) | 6.77 | 0.01804 |
| K (% wt.) | 0.58 (0.02) | 0.7 (0.02) | 12.21 | 0.00259 |
| C:N | 53.4 (1.9) | 47.4 (1.3) | 6.86 | 0.01739 |
| N:P | 8.7 (0.3) | 8.5 (0.3) | 0.25 | 0.62065 |
| C:P | 461 (13) | 401 (18) | 6.84 | 0.01753 |
| N:K | 1.6 (0.1) | 1.5 (0.1) | 1.08 | 0.31251 |
| K:P | 5.5 (0.2) | 5.8 (0.3) | 0.60 | 0.44910 |
| Disaccharides | 687925 (80901) | 675282 (89188) | 0.01 | 0.91754 |
| Hexoses | 15087614 (2434604) | 9348376 (1175812) | 4.51 | 0.04790 |
| Pentoses | 3629391 (425591) | 2813197 (185011) | 3.09 | 0.09560 |
| Deoxyglucose - Deoxygalactose - D-Fucose (S1) | 186401 (20931) | 235288 (47596) | 0.88 | 0.35954 |
| Xylitol – Arabitol (S2) | 4972706 (420033) | 3661582 (343655) | 5.84 | 0.02654 |
| Arginine | 40937 (15001) | 50681 (13679) | 0.23 | 0.63706 |
| Glutamine | 31638656 (4014985) | 53907016 (6633884) | 8.25 | 0.01015 |
| Isoleucine | 86693756 (17314253) | 59725583 (5876334) | 2.18 | 0.15751 |
| Leucine | 189936 (41684) | 292586 (75204) | 1.43 | 0.24804 |
| Methionine | 51280054 (5169907) | 40590246 (6365640) | 1.70 | 0.20882 |
| Phenylalanine | 81321806 (21947781) | 18830053 (4064793) | 7.84 | 0.01184 |
| Proline | 6650984 (1059194) | 7399428 (1062160) | 0.25 | 0.62385 |
| Serine | 2155398 (410865) | 3514112 (257350) | 7.85 | 0.01177 |
| Threonine | 6222604 (805330) | 6516555 (544718) | 0.09 | 0.76586 |
| Tryptophan | 38155778 (9444718) | 40227689 (15161932) | 0.01 | 0.90895 |
| Tyrosine | 9460992 (1140159) | 7127756 (844464) | 2.70 | 0.11743 |
| Valine | 6684223 (778917) | 9159026 (1088263) | 3.42 | 0.08092 |
| Adenine | 16535326 (2280014) | 16399436 (2574516) | 0.00 | 0.96892 |
| Adenosine | 10603069 (581300) | 51089903 (7452674) | 29.33 | 0.00004 |
| Uracil | 10849945 (708900) | 7187568 (795567) | 11.81 | 0.00294 |
| Uridine | 1165314 (144420) | 910312 (227273) | 0.90 | 0.35619 |
| Α-ketoglutaric acid | 90514 (13302) | 414236 (107567) | 8.92 | 0.00791 |
| Citric acid | 27920168 (3498491) | 26207982 (4338792) | 0.09 | 0.76222 |
| Succinic acid | 169452 (22164) | 81687 (12686) | 11.81 | 0.00294 |
| Pyruvic acid | 4166911 (444242) | 3707684 (343914) | 0.67 | 0.42438 |
| Dihydroxytrimethoxyflavone (Flav) | 5241442 (1106237) | 6976702 (1610484) | 0.79 | 0.38618 |
| Catechin | 1514476 (307248) | 2337975 (537068) | 1.77 | 0.19983 |
| Chlorogenic acid | 37103 (11862) | 35879 (4956) | 0.01 | 0.92524 |
| D-pinitol | 1468406 (221329) | 1100968 (189587) | 1.59 | 0.22347 |
| Epigallocatechin | 2418582 (436883) | 4168814 (1110550) | 2.15 | 0.15974 |
| Fumaric acid | 2980847 (335580) | 2283882 (252824) | 2.75 | 0.11448 |
| Kaempferol | 1091990 (189374) | 1103847 (164739) | 0.00 | 0.96284 |
| Luteolin | 13483 (2611) | 19058 (3710) | 1.51 | 0.23496 |
| Myricetin | 504620 (10150) | 282313 (48488) | 20.14 | 0.00028 |
| Protocatechuic acid | 153392 (27099) | 182083 (49499) | 0.26 | 0.61733 |
| Quercetin | 236862 (48000) | 272647 (52616) | 0.25 | 0.62144 |
| Quinic acid | 2175099037 (228895556) | 1401520454 (131567637) | 8.59 | 0.00894 |
| Sodium salicylate | 473715 (75294) | 486469 (63610) | 0.02 | 0.89848 |
| Taxifolin | 2132382 (591347) | 1350087 (298080) | 1.40 | 0.25285 |
| Vanillic acid | 73682 (15631) | 76218 (13928) | 0.01 | 0.90493 |
| Caryophyllene | 134918 (26893) | 98197 (18745) | 1.25 | 0.27736 |
| Sabinene | 291279 (77549) | 253663 (63954) | 0.14 | 0.71262 |
| Thujone | 20058 (3651) | 9043 (1633) | 7.58 | 0.01306 |
| Abscisic acid (ABA) | 848083 (192462) | 444163 (95648) | 3.53 | 0.07648 |
| Choline | 17268377 (2006533) | 19060936 (2702384) | 0.28 | 0.60084 |
| δ-tocopherol | 776842 (113895) | 1243235 (129655) | 7.30 | 0.01457 |
| Gibberellic acid (GA3) | 3303259 (527354) | 3285658 (320919) | 0.00 | 0.97757 |
| Pantothenic acid (Vit B5) | 2512133 (455295) | 3303608 (832949) | 0.70 | 0.41533 |
| Shikimic acid | 13607155 (2263798) | 8847017 (1096036) | 3.58 | 0.07461 |
